# New records of the dolphin *Albertocetus meffordorum* (Odontoceti: Xenorophidae) from the lower Oligocene of South Carolina: Encephalization, sensory anatomy, postcranial morphology, and ontogeny of early odontocetes

**DOI:** 10.1371/journal.pone.0186476

**Published:** 2017-11-08

**Authors:** Robert W. Boessenecker, Erum Ahmed, Jonathan H. Geisler

**Affiliations:** 1 Department of Geology and Environmental Geosciences, College of Charleston, Charleston, South Carolina, United States of America; 2 University of California Museum of Paleontology, University of California, Berkeley, California, United States of America; 3 Department of Anatomy, New York Institute of Technology College of Osteopathic Medicine, Old Westbury, New York, United States of America; Medical University of South Carolina, UNITED STATES

## Abstract

We report five new specimens of xenorophid dolphins from North and South Carolina. Four of the specimens represent the xenorophid *Albertocetus meffordorum*, previously only known from the holotype skull. The other is a fragmentary petrosal from the upper Oligocene Belgrade Formation that we refer to *Echovenator* sp, indicating at least two xenorophids from that unit. Two of the *Albertocetus meffordorum* specimens are from the lower Oligocene Ashley Formation: 1) a partial skeleton with neurocranium, fragmentary mandible, ribs, vertebrae, and chevrons, and 2) an isolated braincase. The partial vertebral column indicates that *Albertocetus* retained the ancestral morphology and locomotory capabilities of basilosaurid archaeocetes, toothed mysticetes, and physeteroids, and caudal vertebrae that are as wide as tall suggest that the caudal peduncle, which occurs in all extant Cetacea, was either wide or lacking. CT data from the isolated braincase were used to generate a digital endocast of the cranial cavity. The estimated EQ of this specimen is relatively high for an Oligocene odontocete, and other aspects of the brain, such as its anteroposterior length and relative size of the temporal lobe, are intermediate in morphology between those of extant cetaceans and terrestrial artiodactyls. Ethmoturbinals are also preserved, and are similar in morphology and number to those described for the Miocene odontocete *Squalodon*. These fossils extend the temporal range of *Albertocetus meffordorum* into the early Oligocene, its geographic range into South Carolina, and expand our paleobiological understanding of the Xenorophidae.

## Introduction

**Fig 1:**
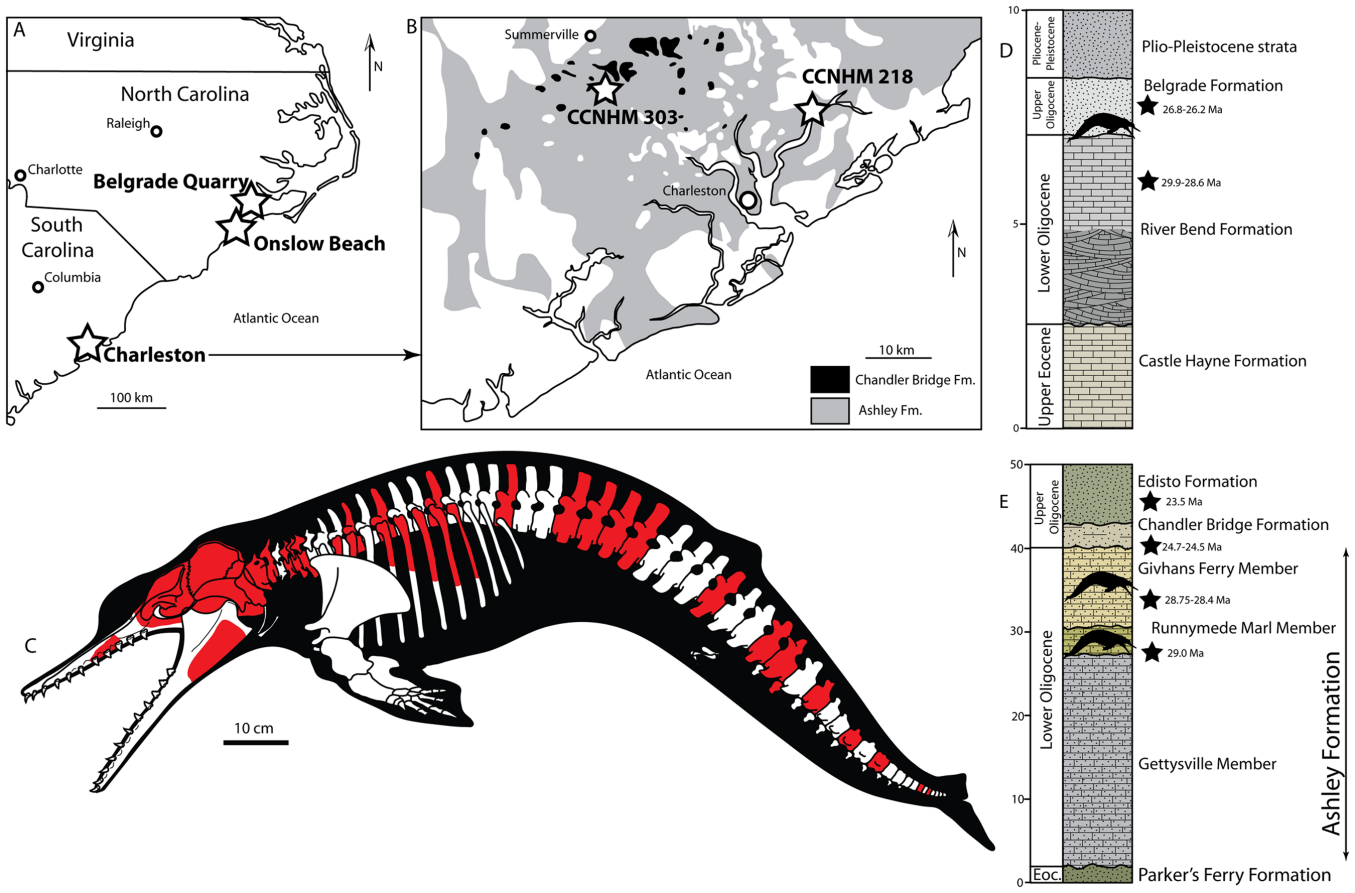
Locality map of occurrences of *Albertocetus meffordorum* in North and South Carolina. (A) and a geologic map of Charleston, South Carolina (B), skeletal reconstruction of *Albertocetus meffordorum* with preserved elements in red (C), generalized stratigraphy at Belgrade Quarry (D) after [29], and generalized Paleogene stratigraphy of the Charleston area (E) after [24, 71]. Gray in geologic map denotes Ashley Formation and black denotes Chandler Bridge Formation. Geologic map simplified and redrawn after Weems and Lewis [26]. ^87^Sr/^86^Sr dates after [24, 31, 32].

Considerable progress has been made in recent years towards elucidating the early evolutionary history of toothed whales (Odontoceti). A series of significant discoveries have revealed that the earliest odontocetes could echolocate [1], possessed inner ear adaptations for ultrasonic hearing [2, 3], and rapidly adapted specialized feeding strategies [4]). Other recent advances have clarified morphological evolution, homology, and phylogeny of early odontocetes [5–10] and others have even referred important new material to taxa with missing holotypes [11]. Like the study of early mysticetes, most studies of Oligocene odontocetes have focused on reporting new taxa based only on a single specimen and only a few have incorporated larger samples ([12]:2). Xenorophid dolphins are the earliest diverging clade within Odontoceti, and demonstrate that echolocation, telescoping (i.e. the posterior migration of the rostral bones and bony nares over the neurocranium), and ecological specialization rapidly evolved shortly after the extinction of the Basilosauridae [1–3]). All prior studies of the Xenorophidae have focused on crania or earbones because the holotypes and only known specimens lack (*Xenorophus sloanii*, *Albertocetus meffordorum*) or have scant (*Cotylocara macei*, *Echovenator sandersi*) postcranial material. This study refers new specimens to *Albertocetus meffordorum* from the upper Oligocene Belgrade Formation and lower Oligocene Ashley Formation (Fig 1A and 1B), and for the first time, one of these specimens has associated postcrania with a cranium and petrotympanics. This new material illuminates virtually unexplored aspects of early odontocete ontogeny and paleobiology including olfaction, encephalization, and locomotion.

## Materials and methods

The partial skeleton CCNHM 303 (Fig 1C) was mechanically prepared by an unknown student preparator; R.W. Boessenecker continued preparation of CCNHM 303 and removed the petrosal. Cemented matrix on the petrosal of CCNHM 303 was removed by acid preparation in dilute (4%) acetic acid. CCNHM 218 was collected under a South Carolina Hobby License issued by the Maritime Research Division (M. Havenstein, pers. comm. 4/2016). Photographs were taken with a Canon Rebel XS and 80mm zoom lens. Measurements were recorded with digital calipers. Terminology for cranial osteology generally follows Mead and Fordyce [13] with some useful additions typically applied to mysticete petrotympanics [14, 15]; postcranial osteological terms after [16].

The *Albertocetus meffordorum* endocast was segmented using the software Amira for Life Sciences (version 5.4.3). A single endocast segment was created using the “magic wand” and “lasso” tools. The cranial cavity was filled with matrix and at times the density of the matrix and bone were similar. In these cases matrix and bone were differentiated by adjusting the contrast gradient and observing the specimen in multiple planes (XY, YZ, and XZ). The finished endocast was smoothed by using the simplifier button under the SurfaceGen module. The total number of faces were decreased by 30% (initial: 18,000 faces; final: 12,600 faces). Final images were taken using snapshot button while rotating the endocast. The SurfaceGen feature in Amira was used to generate an isosurface on the *Albertocetus* endocast. The smoothening of the endocast was left unconstrained to ensure a more accurate final value. Next, the SurfaceArea option was applied to calculate the surface area and volume of the specimen.

The endocranial volumes for *Albertocetus* and other fossil specimens were reduced based on an estimate of non-neural tissue (e.g. meninges, vasculature) within the endocast. To make this estimate we took data on brain mass and brain adnexia in cetaceans from a previous study [17], converted this to volumes using their value of specific gravity for brain tissue of 1.04, log normalized both series, and then calculated a linear regression in the application PAST [18]. These equations allowed us to estimate what fraction of the digital endocast was occupied by the brain and what portion was occupied by adnexia in fossil taxa, specifically those taxa and specimens included in a previous study [19] (S1 Table). One group of extinct cetaceans, the basilosaurids, have an extensive vascular rete that is very visible on an endocast [20, 21]. The above method, which is based on extant taxa, does not account for this morphology. Thus for basilosaurids we used published estimates for the volumes of retia that were derived form detailed study of individual endocasts [21]. Finally we also used this opportunity to update the brain mass estimates for several extant odontocetes in a previous dataset [19] with those from a recent comprehensive study [17].

Body mass was calculated using the following equation from a previous study [19] that relates mass (in grams) to width across the occipital condyles (OCW in mm):

Mass = 1000 x 10^((3.814 x log (OCW)) – 5.345)^. In order to assess the robustness of our body mass estimate we also calculated the body mass of *Albertocetus* and other Oligocene fossil odontocetes using two other equations derived from linear regressions of measurements obtained from extant taxa. The first is an equation that estimates total body length (cm) from the bizygomatic width (BIZYG) of the skull in cm [22]: log(total body length) = 0.92 * (log(BIZYG) – 1.72) + 2.68. This result was then entered into a separate equation that relates body mass to body length [23]: body mass (kg) = 10 ^(2.799^*^(total body length in cm) - 4.464)^.

### Institutional abbreviations

CCNHM, Mace Brown Museum of Natural History, Charleston, South Carolina, USA; ChM, Charleston Museum, Charleston, South Carolina, USA; USNM, National Museum of Natural History, Washington, D.C., USA.

## Results

### Systematic paleontology

Cetacea Brisson, 1762

Pelagiceti Uhen, 2008

Odontoceti Flower, 1867

Xenorophidae Uhen, 2008

*Albertocetus* Uhen, 2008

*Albertocetus meffordorum* Uhen, 2008

#### Referred specimens

**Fig 2:**
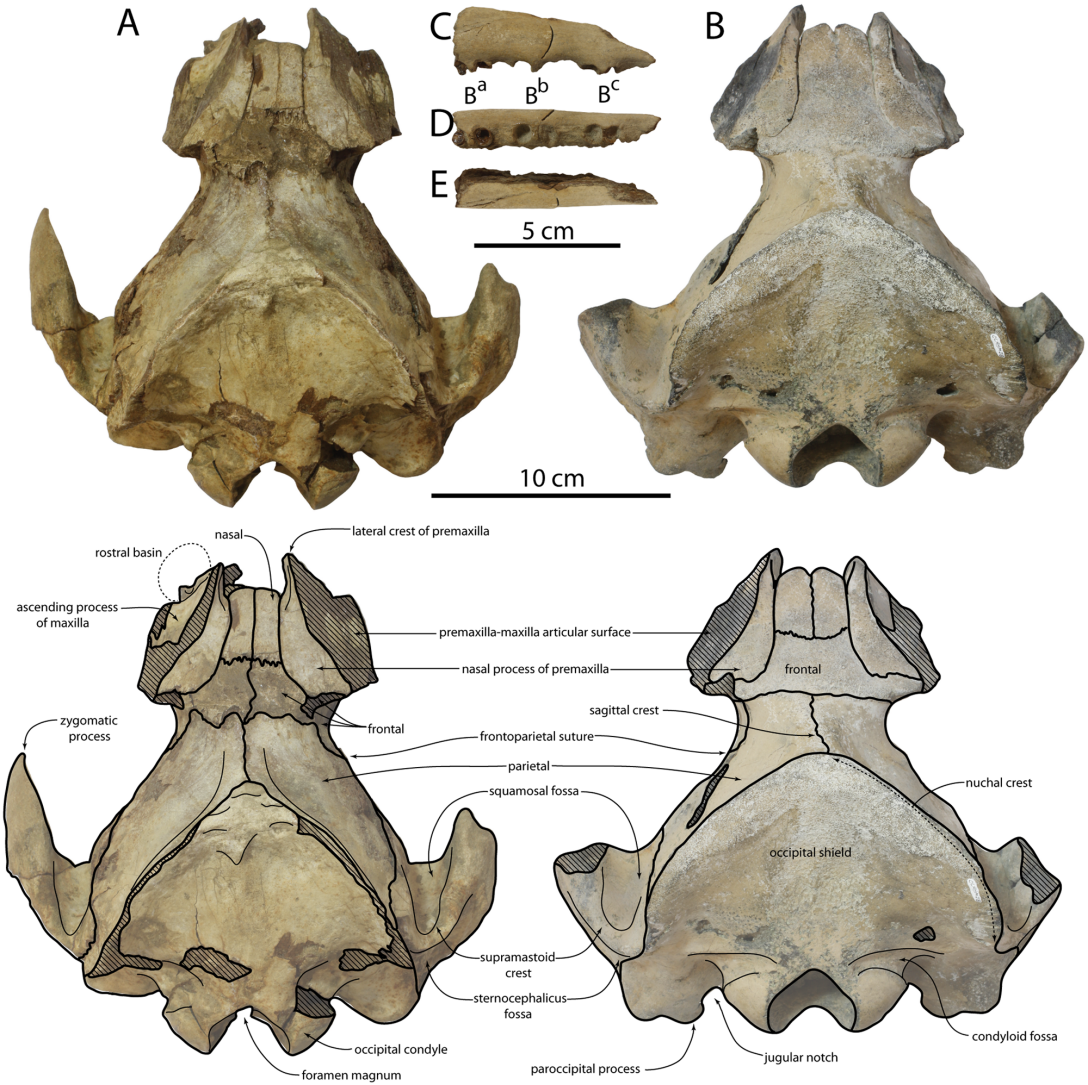
Cranium of *Albertocetus meffordorum*. CCNHM 303 in (A) dorsal view; CCNHM 218 in (B) dorsal view; maxilla fragment of CCNHM 303 in (C) lateral, (D) ventral, and (E) medial view. Cross-hatching denotes damaged or missing bone.

CCNHM 303, partial skeleton (Fig 1C) including partial skull, petrotympanics, atlas, axis, C4, C5, and C6, four thoracic vertebrae, seven lumbar vertebrae, six caudal vertebrae and several ribs, collected sometime before 2010 by an unknown student (J. Carew, pers. comm. 2016) from the Givhans Ferry Member of the Ashley Formation, Dorchester County, South Carolina (Fig 1B). CCNHM 218, partial skull including braincase and left petrosal, collected ex-situ from the Wando River by M. Havenstein (Figs 1B and 2D). Concretionary matrix consists of glauconitic calcarenite consistent with the Runnymede Marl Member of the Ashley Formation. USNM 559392, isolated right petrosal, collected May 2015 by Gary J. Grimsley from the Belgrade Formation, Belgrade Quarry, Jones County, North Carolina (Fig 1B and 1D).

#### Identification

Specimens CCNHM 218 and 303 possess the following synapomorphies of Xenorophidae: 1) nasal process of premaxilla greatly expanded and underlies ascending process of maxilla, 2) premaxilla with lateral crest adjacent to the bony nares, 3) frontal window posterior to orbit with exposure of premaxilla, and 4) large and laterally projecting lateral tuberosity of petrosal [1]. An additional xenorophid feature, a deeply excavated rostral basin [1], appears to be preserved in CCNHM 303. These specimens share with *Albertocetus meffordorum*, to the exclusion of other xenorophids, the following combination of features: 1) retention of a narrow sagittal crest deviating approximately 5–15° anterolaterally to the left (unknown in *Xenorophus sloanii*), 2) triangular apex of occipital shield in dorsal view, 3) nasals rectangular with parallel lateral margins, transverse anterior margin, and dorsally flattened, 4) petrosal with small, circular, pit-like suprameatal fossa (also present in *Xenorophus* sp.). These features indicate that CCNHM 218 and 303 are identifiable as *Albertocetus meffordorum* and not to other named xenorophids.

#### Stratigraphy and age

The Ashley Formation is the oldest of three Oligocene marine units in the Charleston Embayment of South Carolina (Fig 1D). The Ashley Formation predominantly consists of calcarenite unconformably overlying the upper Eocene Parkers Ferry and Harleyville formations; a phosphatic lag occurs at its base [24]. The Ashley Formation includes several phosphatic lags that permit subdivision into three members, from lowest to highest: Gettysville Member, Runnymede Marl Member, and the Givhans Ferry Member [24]. The Ashley Formation is overlain by the patchy, noncalcareous, and richly fossiliferous Chandler Bridge Formation (Fig 1B and 1D) and various younger Neogene units [24]. The Ashley Formation was deposited under nearshore to outer continental shelf depths [25, 26], and a warm temperate climate is indicated by pollen and the presence of billfish [27, 28]. Isolated skull CCNHM 218 was collected *ex situ* from the Wando River with adhering concretionary glauconitic limestone matrix; exposures in this area are mapped as the Runnymede Marl Member of the Ashley Formation [24]. Partial skeleton CCNHM 303 was collected *in situ* from an exposure of the Givhans Ferry Member of the Ashley Formation which has produced other vertebrates including a skeleton of a large, undescribed species of *Xenorophus* (CCNHM 168), an incomplete humerus of the giant bony toothed bird *Pelagornis* sp., cf. *P*. *sandersi* (CCNHM 786), and a partial skull of *Aglyptorhynchus* sp. (CCNHM 1837). Adhering matrix consists of sparsely glauconitic and discontinuously cemented calcarenite; a poorly preserved juvenile odontocete squamosal and fish bones, including a preural vertebra of the blochiid billfish *Aglyptorhynchus*, were found in association with CCNHM 303. Detailed locality information is available on request from CCNHM. The Givhans Ferry Member of the Ashley Formation is lower Oligocene in age and has yielded ^87^Sr/^86^Sr dates of 28.43–28.75 Ma, and the underlying Runnymede Marl Member has yielded a date of 29.0 Ma [24]. An age of 29–28.43 Ma can be applied to fossils of *Albertocetus meffordorum* from the Ashley Formation.

The Belgrade Formation is exposed in various quarries in southeastern North Carolina (Figs 1A and 2C) and consists of a lower unconsolidated gray-brown calcarenite richly fossiliferous with respect to mollusks reflecting shoreface marine deposition (Haywood Landing Member) and an upper finer grained unit with abundant giant oysters (*Crassostrea gigantissima*) reflecting estuarine or possibly shallow marine deposition (Pollocksville Member; [29]). Echinoid assemblages from the Belgrade Formation indicate an upper Oligocene age [30] and *Crassostrea* shells yielded ^87^Sr/^86^Sr dates of 27 ± 1 Ma [31]. Belgrade Quarry has yielded a small vertebrate assemblage from the Belgrade Formation (recorded in CCNHM collections) including cow sharks (*Notorhynchus primigenius*), a nurse shark (*Nebrius* or *Ginglymostoma*), an angel shark (*Squatina* sp.), sand tiger sharks (*Carcharias* sp., *Odontaspis* sp.), megatoothed sharks (*Carcharocles angustidens*), snaggletooth sharks (*Hemipristis serra*), reef and lemon sharks (*Carcharhinus*, *Negaprion*), tiger sharks (*Physogaleus aduncas*, *Galeocerdo casei* or *Galeocerdo mayumbensis*), sawfish (*Anoxypristis* sp.), cownose and bat rays (*Rhinoptera* sp., *Myliobatis* sp.), an extinct devil ray (*Plinthicus stenodon*), a pinfish (*Lagodon* sp.), indeterminate crocodilians and sea turtles, indeterminate odontocete remains, and xenorophid dolphin specimens USNM 559392 (*Albertocetus meffordorum*) and CCNHM 1188 (cf. *Echovenator*) reported in this study. Unfortunately, at Belgrade Quarry collecting is only permitted on spoil piles where specimens are reworked; Pliocene marine vertebrate (*Carcharocles megalodon*, *Carcharodon carcharias*, *Carcharodon hastalis*) and Pleistocene terrestrial mammal (e.g. *Equus*) remains are found occasionally interspersed. However, within the Oligocene section at the quarry studied by [29], marine vertebrate remains are only recorded within the lowermost Belgrade Formation. Limestone cobbles, often containing vertebrate remains (such as the *Albertocetus meffordorum* holotype), washed up on Onslow Beach are assumed to have been eroded from submarine exposures of the Belgrade Formation; ^87^Sr/^86^Sr dates of mollusks from these cobbles indicate an age of 26.5 Ma [10], whereas dates from the Belgrade Formation at Belgrade Quarry range from 27–26.2 Ma [31, 32] At Belgrade Quarry, ^87^Sr/^86^Sr dates from the underlying upper River Bend Formation are 29.9–28.6 Ma [32]. A slightly younger age may be possible for the Belgrade Formation [31, 32], but see [32]. Available dates from the Belgrade Formation in North Carolina thus indicate an age of 26.2–27 Ma [10, 31], late Oligocene.

### Description

#### Premaxilla

The posterior end of the premaxilla extends further posterior than the nasals and has wide contact with the frontal posteriorly and ventrally (Fig 2; Table 1). The premaxilla is transversely and dorsoventrally expanded into a posteriorly widening wedge; approximately the lateral 2/3 of its dorsal surface was originally covered by a dorsolaterally-facing maxilla (fragments remain on both specimens) and the medial third is dorsally flat and widens posteriorly. The medial portion of the premaxilla bears a lateral ridge that runs adjacent to the suture with the maxilla. The premaxilla is transversely narrowest anteriorly adjacent to the anterior end of the nasals where it forms a vertical splint. A shallow parasagittal fissure is present on the dorsolateral surface of the premaxilla where it would have been covered by the ascending process of the maxilla. The premaxilla appears to have been exposed posteroventrally on the roof of the temporal fossa within the "frontal window" [8].

**Table 1:** Cranial measurements of *Albertocetus meffordorum*.

Measurement	CCNHM 303	*CCNHM 218
Bizygomatic width	183.6	-
Bony nares, transverse width	23.2	28.4
Nasals, depth anteriorly	-	12.4
Nasals, anterior width	18.1	22.7
Nasals, maximum width	20.1	26.5
Nasals, maximum length	34.4	34.9
Least interorbital width	60.8	57.2
Separation of nasals/occipital	89.3	88.2
Exoccipital width	138.6	154
Occipital condyle breadth	66.6	70.7
Occipital condyle depth	39.4	42.2
Foramen magnum, transverse width	28.8	31.7
Foramen magnum, dorsoventral depth	27.5	34.2
Width across basioccipital crests	63.2	75
Length of occipital, f. magnum to apex	86.3	86.1
Transverse width of ascending proc. premaxilla	18.7	16.3
Transverse width across ascending premaxillae	71.9	76.9
Anteroposterior length of parietal at midline	31.6	28.1
Anteroposterior length of frontal at midline	23.5	26.7
Deviation of median parietal suture	6.2° to left	23° to left
Maximum width of frontal at vertex	92	74.8

#### Maxilla and dentition

Little of the maxilla is preserved in either specimen except for a rostral fragment of the right maxilla and the base of the rostral portion of the left maxilla (both in CCNHM 303; Fig 2). The rostral fragment is elongate, transversely narrow, anteriorly shallowing in height, and evenly laterally convex in cross section. It preserves alveoli for three double rooted cheek teeth, the roots of the posteriormost of which remain in situ. Judging from the shallowness of the maxilla, these teeth are inferred to be the anteriormost double-rooted postcanines, possibly representing buccal teeth B3-B5 by comparison with *Xenorophus sloanii* [8]. The posterior tooth is missing the crown, but the cross-sections of the broken roots exhibit completely closed pulp cavities. The base of the rostral section of the maxilla is longitudinally “stepped”, with a transverse vertical face at the level of the antorbital notch separating the facial and rostral sections and representing the posterior wall of the rostral basin (= antorbital fossa of [8]). Several dorsal infraorbital foramina are present here but obscured by matrix. Posteriorly, the ascending process of the maxilla forms a thin sheet that overlies the premaxilla.

#### Nasal

The nasals are approximately rectangular in dorsal view and dorsally flat; the nasals, along with the medial portion of the frontal, lack the postnarial fossa developed in *Echovenator* and *Cotylocara* (Fig 2; Table 1). In CCNHM 218, this region is even slightly convex. The nasals in CCNHM 303 are somewhat narrower than in CCNHM 218, possibly reflecting ontogenetic differences. The posterior margin of the nasals is nearly transverse; in CCNHM 218 there is a slight triangular wedge of frontal penetrating anteriorly between the nasal and premaxilla, but not as extreme as in *Albertocetus meffordorum* or *Xenorophus sloanii*. The frontonasal suture is clear in CCNHM 303 but anastomosing and nearly completely closed in CCNHM 218. The nasals are dorsoventrally deep and appear to deepen posteriorly.

#### Frontal

A narrow band of frontal is exposed dorsally, forming a trapezoidal shape and extending anteriorly between the nasal processes of the premaxillae to meet the nasals (Fig 2; Table 1). Similar to the nasal, the frontal is dorsomedially flattened, and even somewhat convex in CCNHM 218; both specimens lack a postnarial fossa as present in *Echovenator* and *Cotylocara*. A thin lateral sheet of frontal posterior to the nasal process of the premaxilla is missing on both sides in CCNHM 303 but present on the right side in CCNHM 218; this sheet posteriorly covers the medial portion of the premaxilla, but the remainder of the posterior portion of the premaxilla would have been exposed ventrally on the temporal fossa (see above). Ventrally, the frontal forms the anteromedial part of the temporal fossa. The ventral part of the frontoparietal suture is vertical but as it ascends it curves anteriorly to form a sigmoidal curve towards the orbitotemporal crest. Though damaged in CCNHM 303, the infraorbital region is well-preserved in CCNHM 218, where it exhibits a large, deep, and anterolaterally curving frontal groove. The canal emanates from the large orbital fissure; the orbital fissure is possibly taphonomically enlarged in both specimens. The frontal forms the anterior margin of the orbital fissure.

#### Parietal

**Fig 3:**
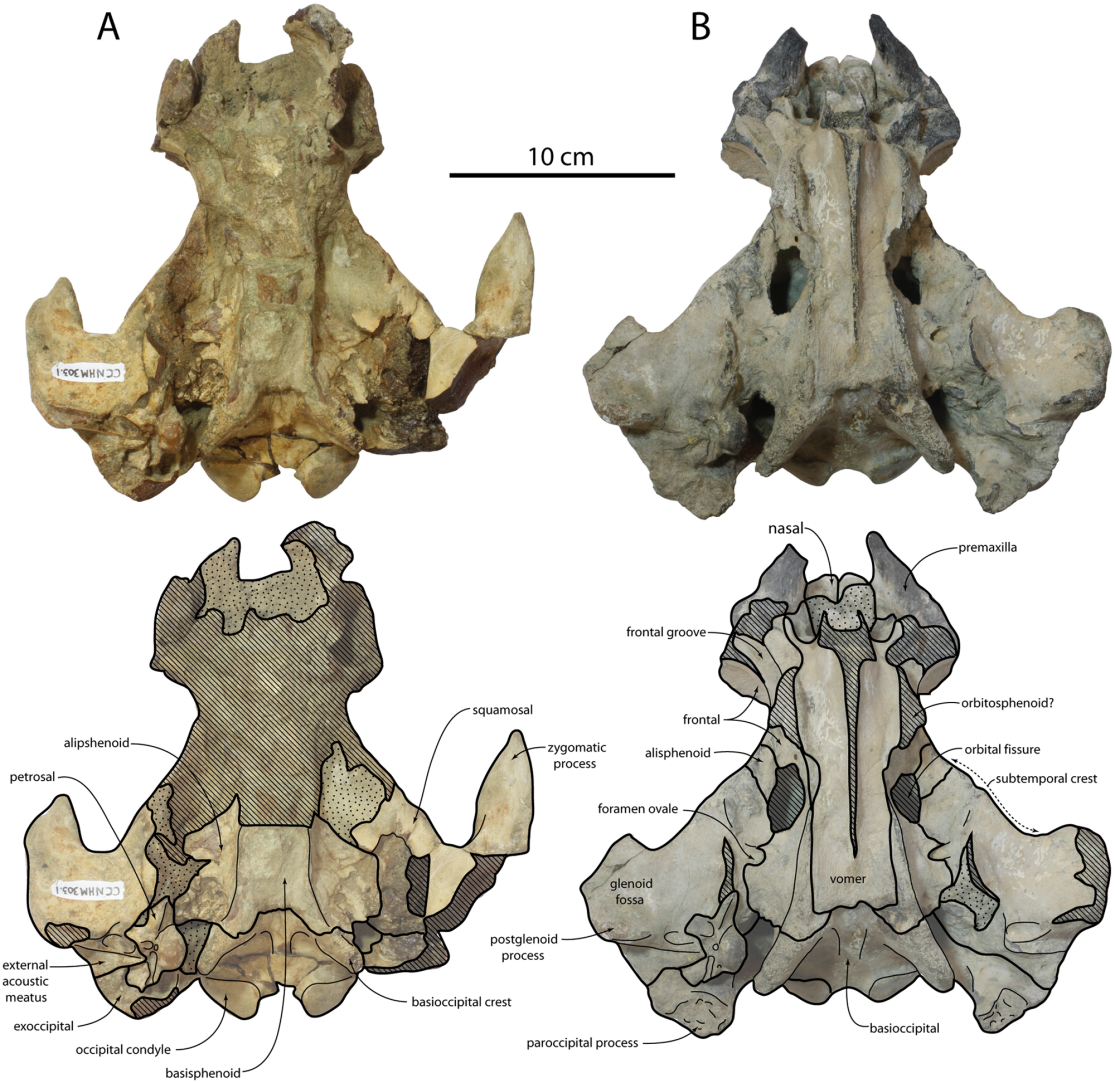
Cranium of *Albertocetus meffordorum* in ventral view; CCNHM 303 (A) and CCNHM 218 (B).

**Fig 4:**
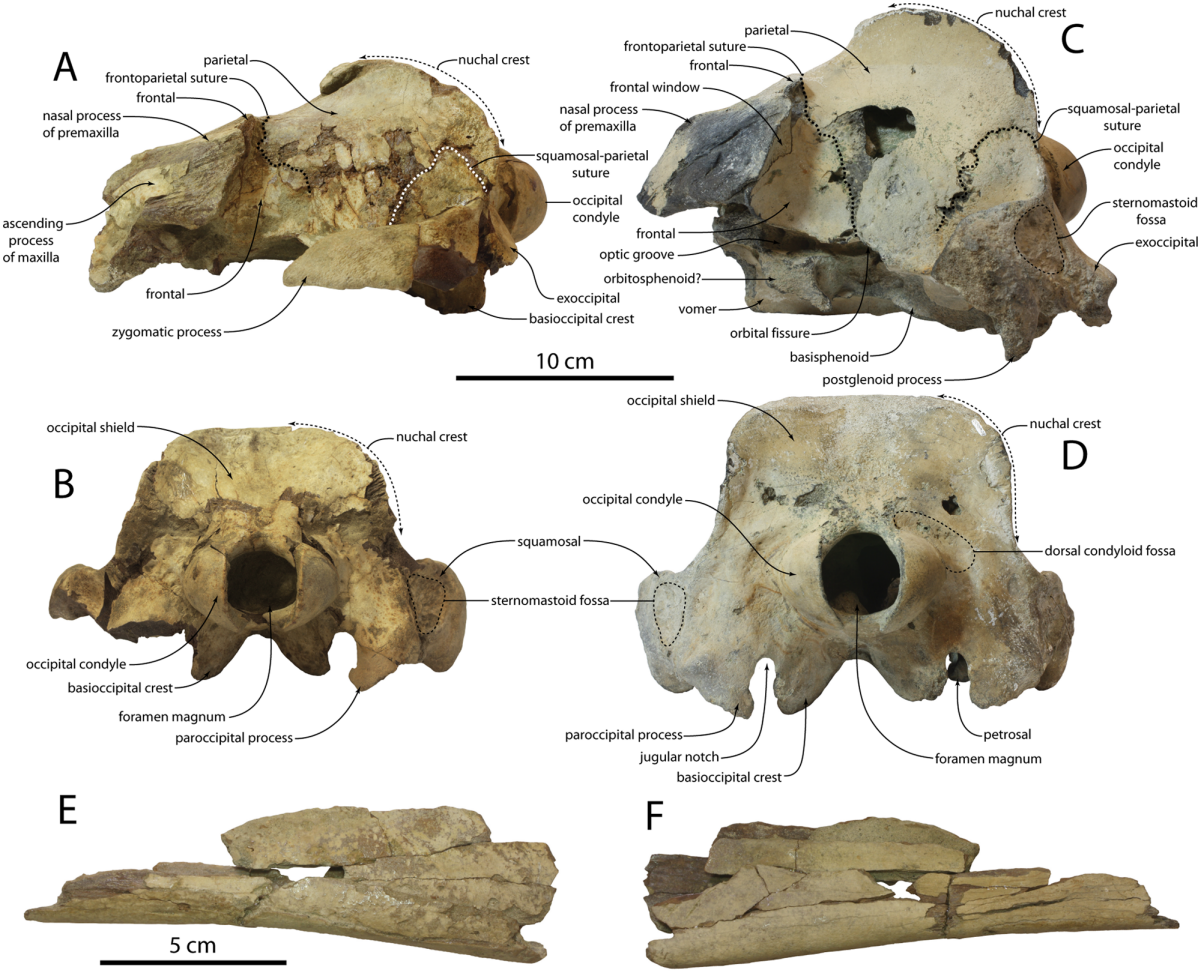
Cranium and mandible of *Albertocetus meffordorum*. CCNHM 303 in (A) left lateral view and (B) posterior view; CCNHM 218 in (C) left lateral and (D) posterior view. Mandible in (E) lateral and (F) medial view.

The parietal forms most of the gently convex lateral wall of the braincase (Figs 2 and 3; Table 1). The parietals contact medially along the vertex to form a low sagittal crest which deviates anterolaterally to the left side; in CCNHM 218, the sagittal crest deviates 14° to the left from the sagittal plane. This indicates that this morphology in the holotype is representative of the species, not taphonomic in origin. This asymmetry is shared with undescribed specimens of *Echovenator* (CCNHM 217, 219), but appears to be absent in *Cotylocara macei* and *Echovenator sandersi*. The median parietal suture is incompletely closed in both skulls, particularly in CCNHM 303 (Fig 2). In CCNHM 303 a posterolaterally directed sulcus is developed parallel with the temporal line ([13]:78), potentially reflecting an incompletely closed parietal-occipital suture; it is completely closed in CCNHM 218. The nuchal crest is low in CCNHM 303 but relatively higher in CCNHM 218 (Figs 2 and 4). The parietal extends ventrally between the frontal and squamosal and contributes to a weak subtemporal crest; it shares a sinuous suture with the squamosal. Dorsally, the suture curves posteroventrally, giving the parietal a posterior hook-like shape that curves around the dorsal margin of the squamosal. A small triangular knob encircled by a shallow sulcus at the apex of the occipital shield may reflect the interparietal.

#### Occipital

**Fig 5:**
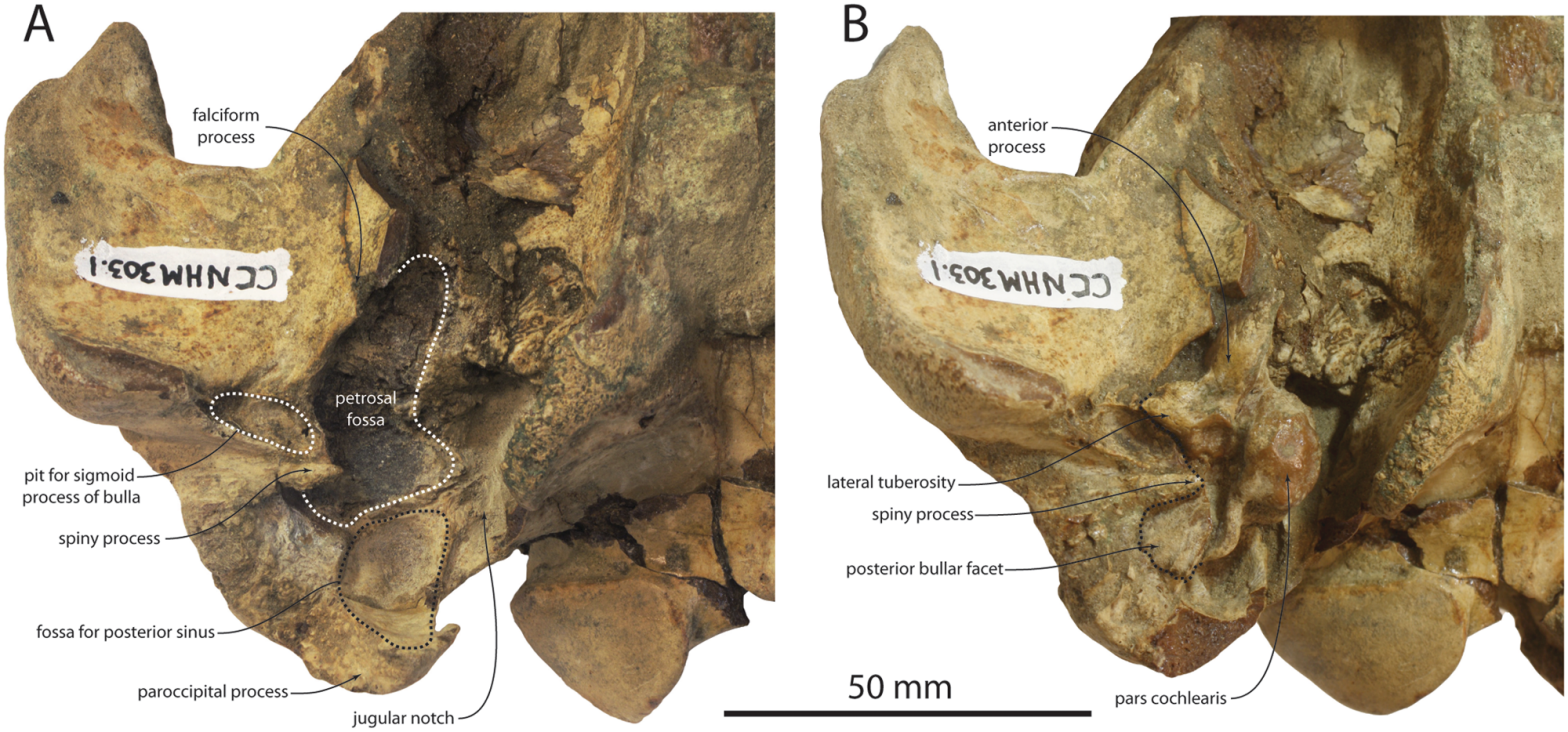
Basicranium of *Albertocetus meffordorum* (CCNHM 303) in ventral view, after (A) and prior to (B) removal of the petrosal and attachment of the paroccipital process.

The occipital shield is semicircular in posterior view and steeply sloping; in dorsal view the shield is triangular at its apex, and somewhat more rounded in CCNHM 218 (Figs 2–4; Table 1). No external occipital crest is developed but on CCNHM 303 a slight nuchal tubercle is present anterodorsally; the shield is otherwise nearly flat to slightly concave. The occipital condyle is small, set on a short pedicle, and surrounded by a deep crescentic condyloid fossa. The dorsal and ventral condyloid fossae are laterally contiguous. In CCNHM 303 the fossae are pitted. The exoccipital faces posterodorsally and descends posteroventrally towards a large tubercle at its ventrolateral extremity. The paroccipital process is damaged in CCNHM 303 but well preserved in CCNHM 218, where it is developed as an elongate, triangular, and ventromedially-curving process that extends ventral to the basioccipital crest (Figs 4 and 5A). The paroccipital process is separated from the basioccipital crest by a deep jugular notch. Two fossae are present on the ventral edge of the exoccipital: the first is a small, rugose pit located at the ventral apex of the paroccipital process, and the second is a concave, smooth pit within the petrosal fossa positioned immediately posterodorsal to the pars cochlearis of the petrosal (Fig 5). The first has been tenuously identified as the origin for the stylohyoid cartilage [12], whereas the second (= deep fossa of [1]) may be homologous to the fossa for the posterior sinus [33]. The basioccipital crests are prominent, posteriorly diverging, and somewhat transversely thickened, broadly similar to the condition in archaic mysticetes like Aetiocetidae.

#### Squamosal

The left squamosal of CCNHM 303 bears a complete zygomatic process but is missing the postglenoid process, whereas the opposite is true for the right squamosal (Fig 2; Table 1). The zygomatic is elongate with a sinuous dorsal margin and a concave ventral margin; in lateral view the anterior end of the zygomatic process is pinched into an acute apex. The supramastoid crest is developed as a low ridge, and situated lateral to the anteroposteriorly short but deep squamosal fossa. Posterolaterally the squamosal portion of the subtemporal crest becomes sharper and more strongly defined than the portion formed by the parietal.

In lateral view, a shallow but rugose sternomastoid fossa is occurs on the posterior margin of the zygomatic process (Fig 4). It faces posterolaterally and is confined to the squamosal, unlike *Echovenator*, where it extends posteromedially onto the exoccipital. The glenoid fossa is transversely broad and shallowly concave with indistinct limits. Medial to the glenoid fossa the falciform process descends ventromedially to underlap the ventrolateral side of the anterior process of the petrosal. The apex of the postglenoid process projects anteroventrally and is continuous medially with a sharp anterior meatal crest that partially defines a deep oval pit for the sigmoid process of the tympanic bulla. A deep pit (= periotic fossa of [34] is present for the petrosal within the petrosal fossa (Fig 5A); dorsal to this the squamosal is very rugose. The pit for the petrosal (sensu [14]) is bilobate, with a small pit anteriorly for the anterodorsal angle and a larger posterior pit to receive the posterodorsal angle. The sharp posterior meatal crest defines the external acoustic meatus and leads to an acute spiny process; the spiny process is set about 2 mm ventral to the posterior bullar facet. A low post-tympanic process extends laterally from the posterior meatal crest and excludes the posterior process of the petrosal from the lateral wall of the braincase (Fig 5B)–indicating the braincase was amastoid. Anteriorly, the squamosal forms the lateral margin of the foramen ovale and contributes to the lateral margin of the orbital fissure.

#### Vomer, sphenoid, and ethmoid

The vomer extends posteriorly almost to the anterior margin of the basioccipital crests, and in the process underlaps the pre- and basisphenoid (Fig 3). Anterior to the orbital fissure the choanae are well formed and begin to rise dorsally; they are not vertical owing to the anterior placement of the nares. The presphenoid is only exposed within the cross section of the broken vomer. The orbitosphenoid was possibly exposed ventral to the optic groove and appears to contribute to the medial margin of the orbital fissure. The alisphenoid does not appear to be exposed laterally in the temporal fossa, and forms the dorsomedial wall of a trough between the petrosal fossa and pterygoid fossa. In CCNHM 218 the alisphenoid forms the posteromedial margin of the orbital fissure and a narrow anterolaterally oriented strip of alisphenoid extends from the anterolateral margin of the periotic fossa. The ethmoid recess is large and exhibits some turbinates, but is filled with matrix (see Nasal Cavities and Turbinates). The vomer bears many branching vascular grooves within the internal choanae.

#### Nasal cavities and turbinates

**Fig 6:**
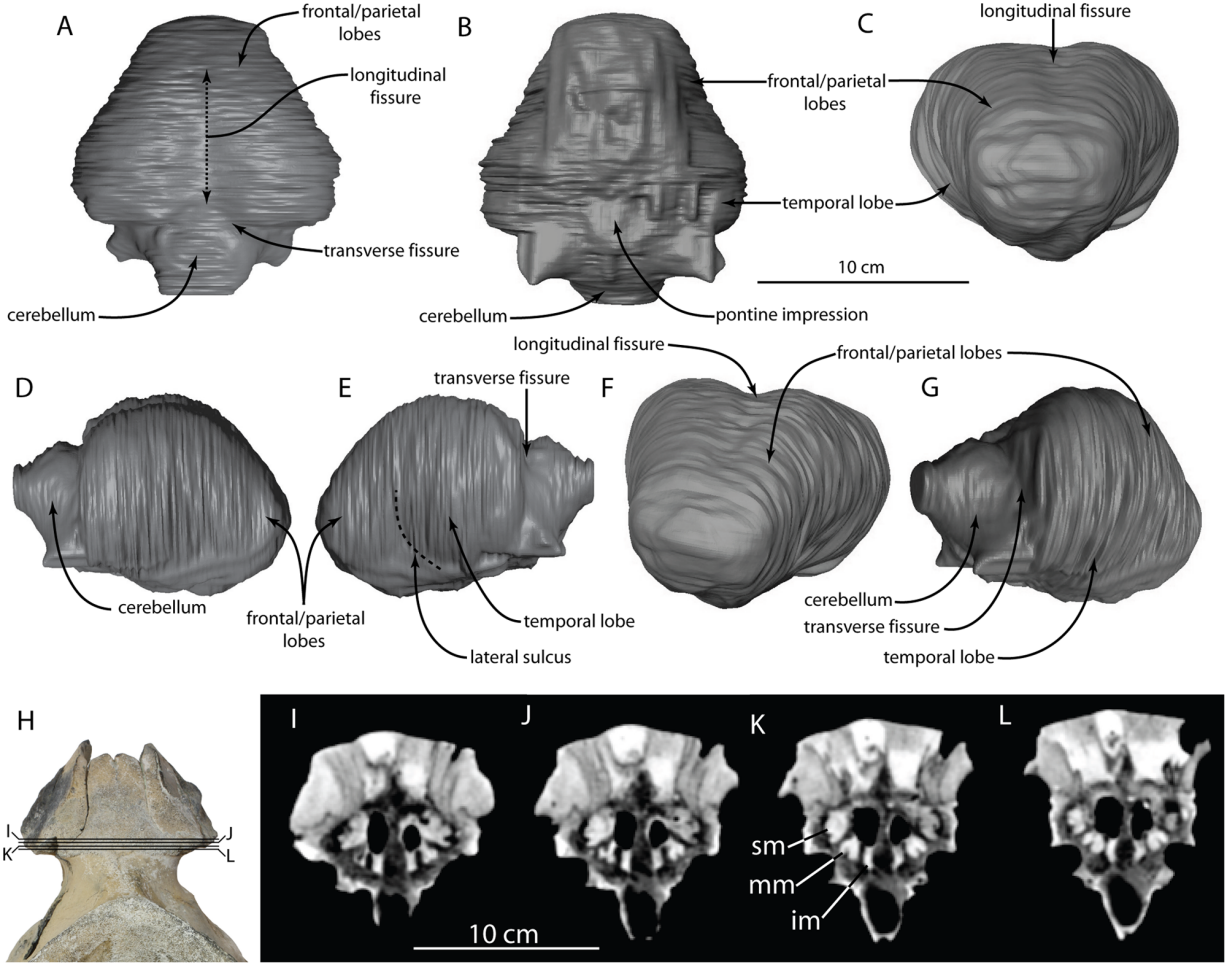
*Albertocetus meffordorum* endocast (CCNHM 218) in (A) dorsal, (B) ventral, (C) anterior, (D) right lateral, (E) left lateral, (F) anterodorsolateral, and (G) posterolateral views; (H), photograph of skull showing positions of CT slices; (I-L) CT slices of interorbital region showing ethmoid labyrinth. Abbreviations: IM, Inferior Meatus; MM, Middle Meatus; SM, Superior Meatus.

A portion of the nasal turbinates are preserved. There appears to be three distinct ethmoturbinals, each separated by a nasal meatus (Fig 6I–6L). We here use the term superior, middle, and inferior meatus, although the structures in *Albertocetus* referred to as middle and inferior meatus are unlikely to be homologous to those structures in humans [35]. Each turbinal is an uncoiled, thick, longitudinal ridge, much like the morphology seen in a specimen of *Squalodon* from the Miocene Calvert Cliffs [36]. Interestingly, that specimen has a comparable number of ethmoturbinals (3 to 4), and based on some phylogenies that place xenorophids and *Squalodon* outside the odontocete crown group (e.g. [37]), we can speculate that this number is primitive for crown Odontoceti.

#### Cranial endocast

The endocast of the cranial cavity is smooth (Fig 6A–6G), suggesting that the meninges are quite thick. There is a clear division noted between the two hemispheres where the dorsal sagittal sinus is hypothesized to be present, although there is no trace of the latter on the endocast (Fig 6A and 6F). The two cerebral hemispheres are symmetric and equal in volume. The cerebrum enlarges towards its posterior end (Fig 6A), and there is no evidence of an olfactory bulb, tract, or pyriform lobe. This contrasts sharply with the large piriform lobe and olfactory bulbs of extant and extinct artiodactyls [38]. While extant cetaceans have spherical brains [39], the brain in *Albertocetus* is more elongate and intermediate in morphology between those of the terrestrial artiodactyls, such as *Diacodexis ilicis* [38], and those of extant cetaceans. The overall shape is reminiscent of basilosaurid archaeocetes, yet differs in possessing proportionally much larger cerebral hemispheres [20, 21, 40]; the endocast compares more favorably with the archaic odontocete *Prosqualodon davidis* [40]. The endocast of *Albertocetus* is more anteroposteriorly compressed than a figured, but undescribed, endocast of another species of xenorophid represented by ChM PV4266 ([21]: Fig 6).

Most other gross anatomical divisions of the brain are not clear in the endocast of *Albertocetus*. The two exceptions are clear sulci separating the cerebral hemispheres from the cerebellum and a lateral sulcus (best visible on the left side; Fig 6E) that separated a larger temporal lobe from a smaller undifferentiated frontal and parietal lobes. By contrast, the combined frontal and parietal lobes have greater [41] or subequal surface area to the temporal lobes in extant cetaceans [39, 42, 43]. This may suggest that the temporal lobe increased in size prior to the enlargement of the frontal and parietal lobes; however, the endocasts of other Oligocene odontocetes need to be described before this suggestion can be adequately tested.

At the anteriormost aspect of the endocast, we expected to see a cribriform plate marking the division between the nasal and cranial cavities; however, there was no trace of the cribriform plate despite this structure being present in the Miocene odontocete *Squalodon* [36]. A cribriform plate has not been observed in other xenorophids (Geisler, pers. obs.), thus it is unclear if its absence is taphonomic or anatomically genuine.

#### Petrosal

**Fig 7:**
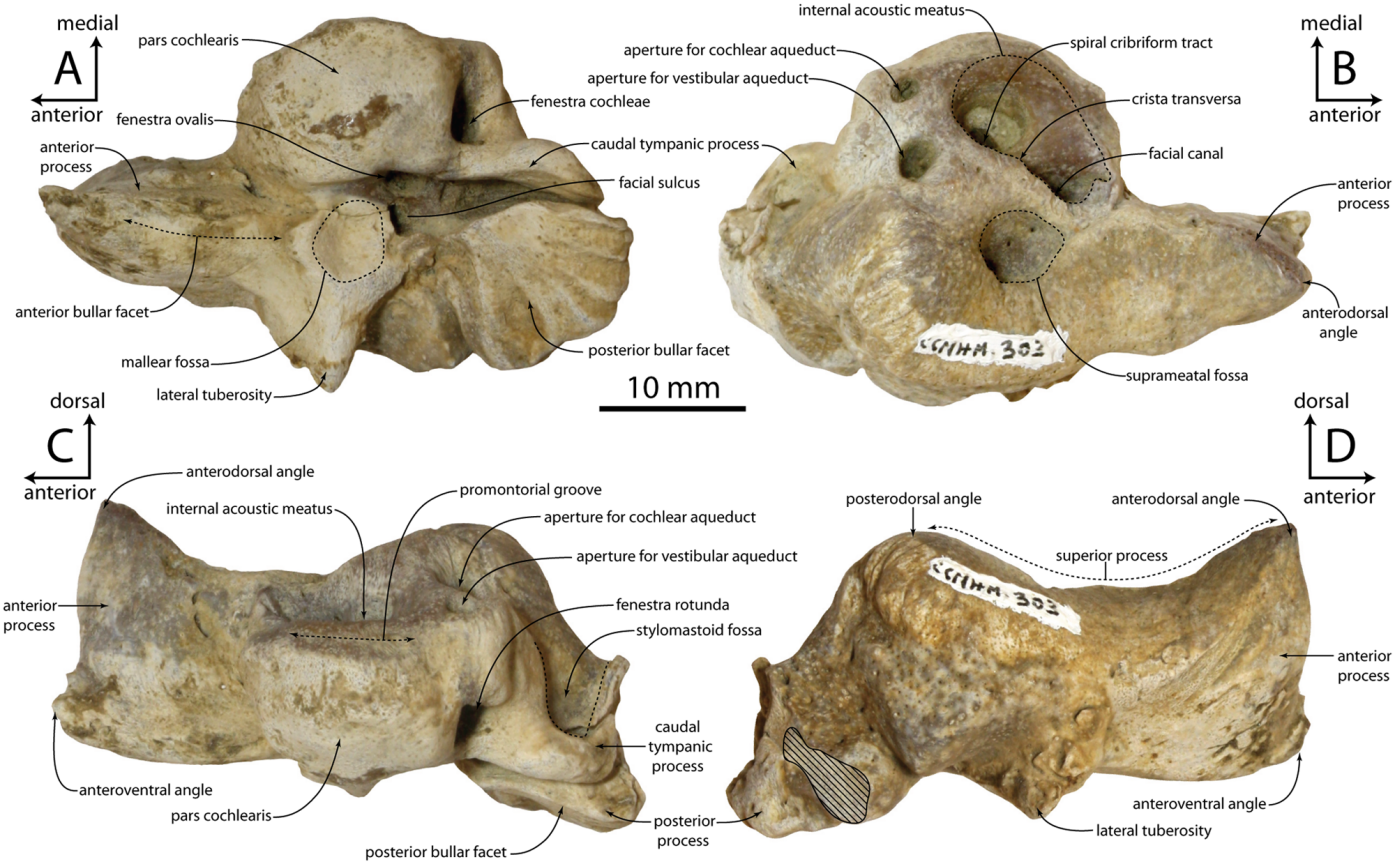
Petrosal of *Albertocetus meffordorum* (CCNHM 303) in ventral (A), dorsal (B), medial (C), and lateral (D) view.

The petrosal is compact with a very large pars cochlearis, short posterior process, a long hatchet-shaped anterior process, and an enlarged and triangular lateral tuberosity (Fig 7; Table 2). The lateral side of the anterior process is convex, its medial side flat, and the anterior bullar facet on its ventral side is indistinct. The anterior process exhibits a spur-like anterodorsal angle; the angle is posteriorly contiguous with a dorsally concave superior process as in *Cotylocara* and *Echovenator*. The anterior incisure opens at a 90° angle. The lateral tuberosity is oriented at a 90° angle to the anterior process and is triangular in outline, unlike *Cotylocara* where it is rectangular; medially it preserves a large, deep mallear fossa with a raised rim. The pars cochlearis is large, hemispherical with an evenly rounded anteromedial margin. A large posteromedial bulge is present dorsal to the fenestra cochleae. A fine, shallow promontorial groove occurs immediately ventral to the medial rim of the internal acoustic meatus. The caudal tympanic process is elongate, nearly as long as the posterior bullar facet, and closely approaches it. The bullar facet is flat with some grooves, subquadrate in shape, and proportionally small (Fig 7A). Relative to promontorial length, the posterior bullar facet (80% in CCNHM 303) is similar to *Echovenator* (76%) and proportionally larger than in *Cotylocara* (47%). A delicate vertical ridge extends dorsal to the posterior bullar facet and clearly separates the stylomastoid fossa from the facial sulcus.

**Table 2:** Petrosal measurements of *Albertocetus meffordorum*.

Measurement	CCNHM 303	CCNHM 218	USNM 559392
Anteroposterior length	39.9	41	42.2
Promontorial width	13.4	15.2	13.6
Pars cochlearis, ant-post length	18.9	18.6	19.1
Pars cochlearis, dorsoventral depth	10.2	10.7	13.4
Anterior process, anteroposterior length	11.8	13.8	15.1
Anterior process, transverse width	8.1	8.6	7.9
Anterior process, dorsoventral depth	10.3	-	10.6
Separation between fenestra rotunda and cochlear aqueduct	6.4	-	5.8
Separation between fenestra cochleae and vestibular aqueduct	7	-	6.9
Separation between fenestra ovalis and fenestra cochleae	3.4	-	3.5
Posterior bullar facet, anteroposterior length	10.7	-	10.2
Posterior bullar facet, transverse width	10.7	10.6	15
Internal acoustic meatus, maximum length	10.2	11	13.7
Internal acoustic meatus, transverse width	7.1	-	10.7
Suprameatal fossa, anteroposterior length	5.4	-	7.5
Suprameatal fossa, transverse width	4.6	-	3.5

The dorsal apertures for the facial canal, foramen singulare, and spiral cribriform tract are recessed into an ovoid internal acoustic meatus (Fig 7B). The aperture for the facial canal is circular; the crista transversa and crest between the foramen singulare and spiral cribriform tract are of similar height. The oval, transversely narrow foramen singulare is smaller than the facial canal; the spiral cribriform tract is circular and larger in diameter than the facial canal. The facial canal is positioned anterolateral to the spiral cribriform tract, and the opening of the foramen singulare is positioned level to the anterior half of the spiral cribriform tract. A low, bulbous tuberosity (= pyramidal process of [44]) is situated between the internal acoustic meatus, suprameatal fossa, and aperture for the vestibular aqueduct; a tall, blunt ridge extends anteriorly from this tuberosity and separates the meatus from the suprameatal fossa. The oval aperture for the cochlear aqueduct is slightly smaller than the circular aperture for the vestibular aqueduct. The suprameatal fossa is developed as a small, circular pit that is much smaller than the internal acoustic meatus; a sulcus emanates anteromedially from it (Fig 7B). Fine creases emanate radially from the edges of the suprameatal fossa. The superior process is low and smooth, and forms a distinct edge lateral to the suprameatal fossa. The posterodorsal angle (Boessenecker and Fordyce, 2014: 118) is convex and bulbous.

**Fig 8:**
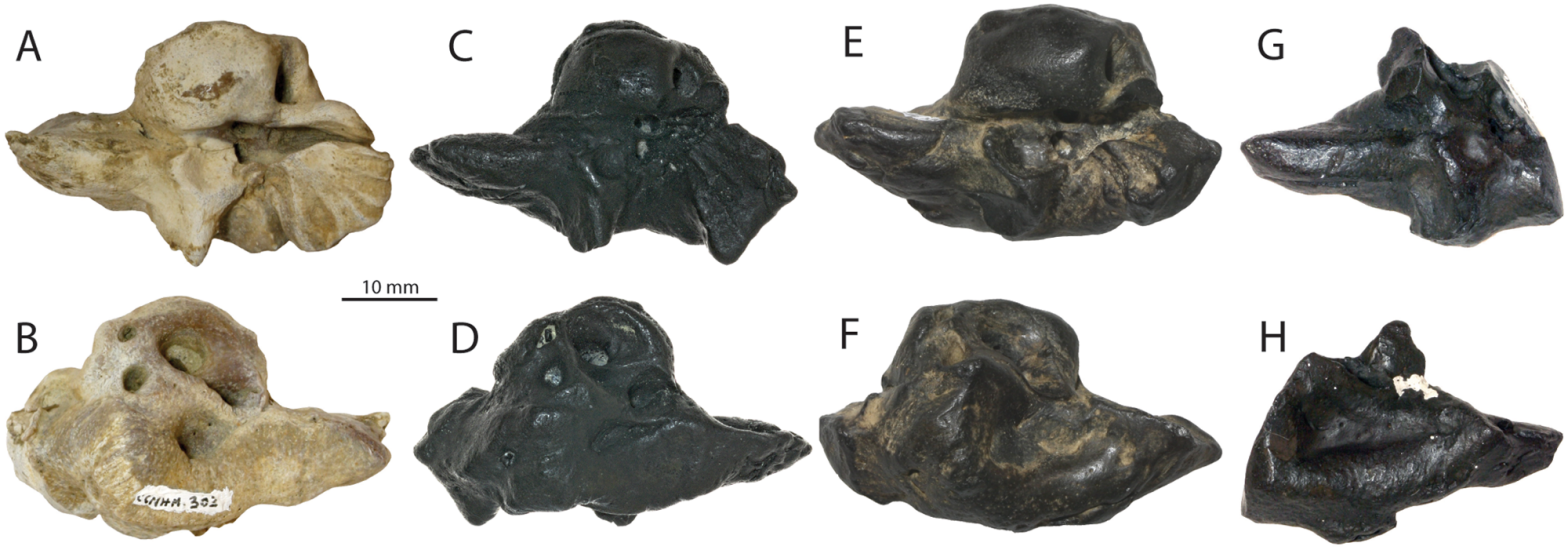
Petrosals of *Albertocetus meffordorum* and *Echovenator* sp. from the Ashley Formation and Belgrade Formation of South and North Carolina (respectively). *Albertocetus meffordorum*, CCNHM 303 (Ashley Fm., SC) in ventral (A) and dorsal (B) view, and USNM 559392 (Belgrade Fm., NC) in ventral (C) and dorsal (D) view; *Echovenator* sp., USNM 534010 (Belgrade Fm., NC) in ventral (E) and dorsal (F) view, and CCNHM 1188 (Belgrade Fm., NC) in ventral (G) and dorsal (H) view.

An isolated petrosal (USNM 559392; Fig 8C and 8D) from the upper Oligocene Belgrade Formation of North Carolina is referable to *Albertocetus meffordorum* on the basis of several shared features with CCNHM 303, including a small, circular, pit-like suprameatal fossa, bulbous posterodorsal angle with radially oriented sulci, and an equidimensional, quadrate posterior bullar facet; none of these features are visible in the holotype petrosal. USNM 559392 differs from CCNHM 303 in a few minor ways, including possessing a deeper groove for the tensor tympani, a more shallowly incised crease anterolateral to the posterior bullar facet, and a dorsoventrally lower and transversely less inflated superior process and posterodorsal angle. Other differences (shape of the anterior process, posterior process) are attributable to damage in these specimens.

These petrosals of *Albertocetus* (CCNHM 303, USNM 559392; Figs 7 and 8C and 8D) share the aforementioned features with *Xenorophus* sp. (CCNHM PV 7677, CCMNH 104; Geisler and Sanders, 2003; Sanders and Geisler, 2015), but differ from *Xenorophus* sp. in their smaller size, proportionally smaller pars cochlearis, longer anterior process, and absence of a longitudinal ridge on the ventral surface of the pars cochlearis. In *Cotylocara* and *Echovenator*, the suprameatal fossa is transversely wider and anteroposteriorly longer than in CCNHM 303, and the fossa is relatively larger than the internal acoustic meatus; faint sulci radiating from the suprameatal fossa are also present in *Cotylocara* and *Echovenator*.

#### Tympanic bulla

**Fig 9:**
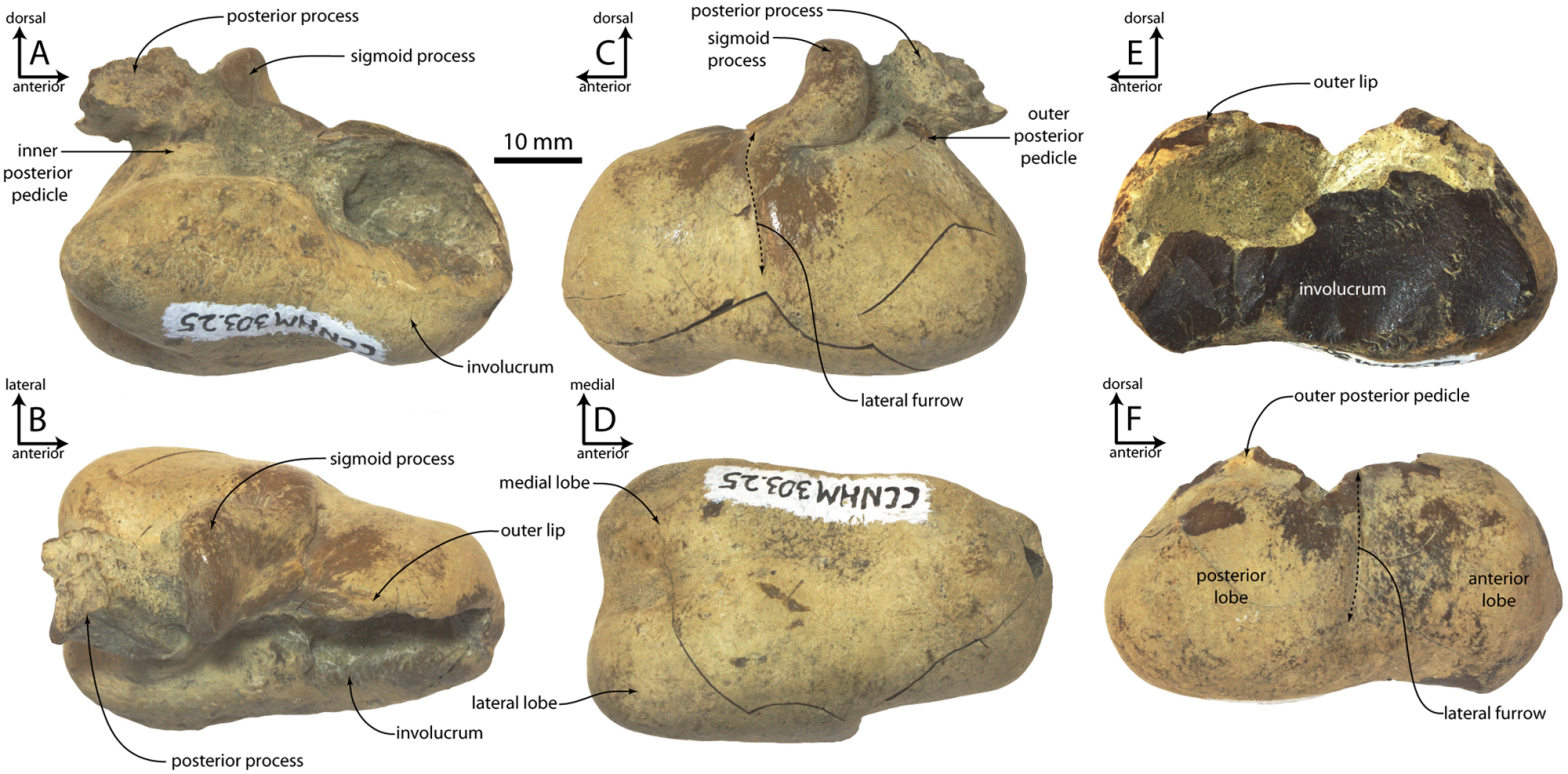
Tympanic bullae of *Albertocetus meffordorum* (CCNHM 303). Left bulla in (A) medial, (B) dorsal, (C) lateral, and (D) medial view; right bulla in (E) medial and (F) lateral view.

The tympanic bulla is subrectangular in dorsal and ventral views, and the bulla anterior to the lateral furrow (= anterior lobe of [15]) is much narrower than that posterior to it (Fig 9; Table 3). The involucrum is bilobate in medial view (Fig 9A) and dorsoventrally pinched at the approximate position of the lateral furrow; a “stepped” dorsal margin of the involucrum is shared with basilosaurids, toothed mysticetes [45], eurhinodelphinids, and ziphiids. The median furrow is shallow and anteroposteriorly short, separates subequal lateral and medial posterior prominences ventrally (Fig 9D). An additional shallow fossa is present anterior to the median furrow. The outer posterior prominence projects far posteroventrally in lateral view, projecting further posteriorly than the posterior process. The posterior process is small with a subtriangular articular facet, and it extends nearly as far dorsally as the sigmoid process. The posterior surface of the posterior process of the bulla is rugose and pitted. The inner posterior pedicle is swollen. Matrix obscures other details of the elliptical foramen and inner and outer posterior pedicles, conical process, and tympanic cavity. The sigmoid process is rectangular in dorsal view, transversely oriented, and positioned approximately 1/3 of the distance from the posterior to anterior margin; in lateral view the sigmoid fissure forms a horizontal cleft as in other Odontoceti. The outer lip is broadly convex laterally and bears a shallow lateral furrow that trends anterodorsally, dividing the bulla into a somewhat shorter anterior lobe and slightly longer posterior lobe.

**Table 3:** Tympanic bulla measurements of *Albertocetus meffordorum*.

Measurement	CCNHM 303
Anteroposterior length	51.8
Transverse width of bulla at sigmoid process	30.2
Maximum width posterior lobe	32.2
Maximum length anterior lobe	22.7
Maximum length posterior lobe	29.2

#### Mandible

The posterior part of a fragmentary right mandible is preserved but lacks the coronoid process and mandibular condyle; the mandibular walls are thin and a large mandibular foramen was present (Fig 4E and 4F) No margins of the mandibular foramen are preserved.

#### Atlas

**Fig 10:**
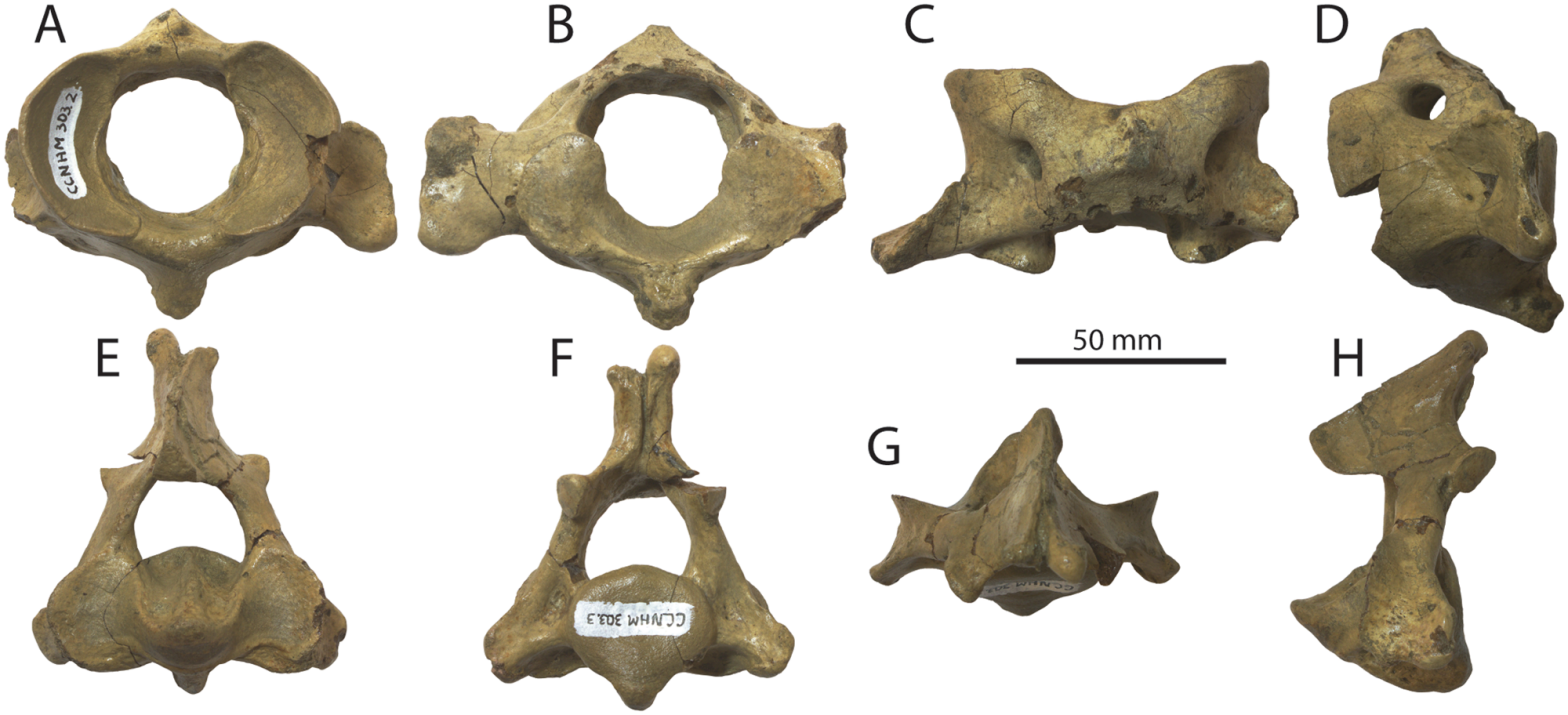
Atlas and axis of *Albertocetus meffordorum* (CCNHM 303). Atlas in (A) anterior, (B) posterior, (C) dorsal, and (D) left lateral view; Axis in (E) anterior, (F) posterior, (G) dorsal, and (H) left lateral view.

**Fig 11:**
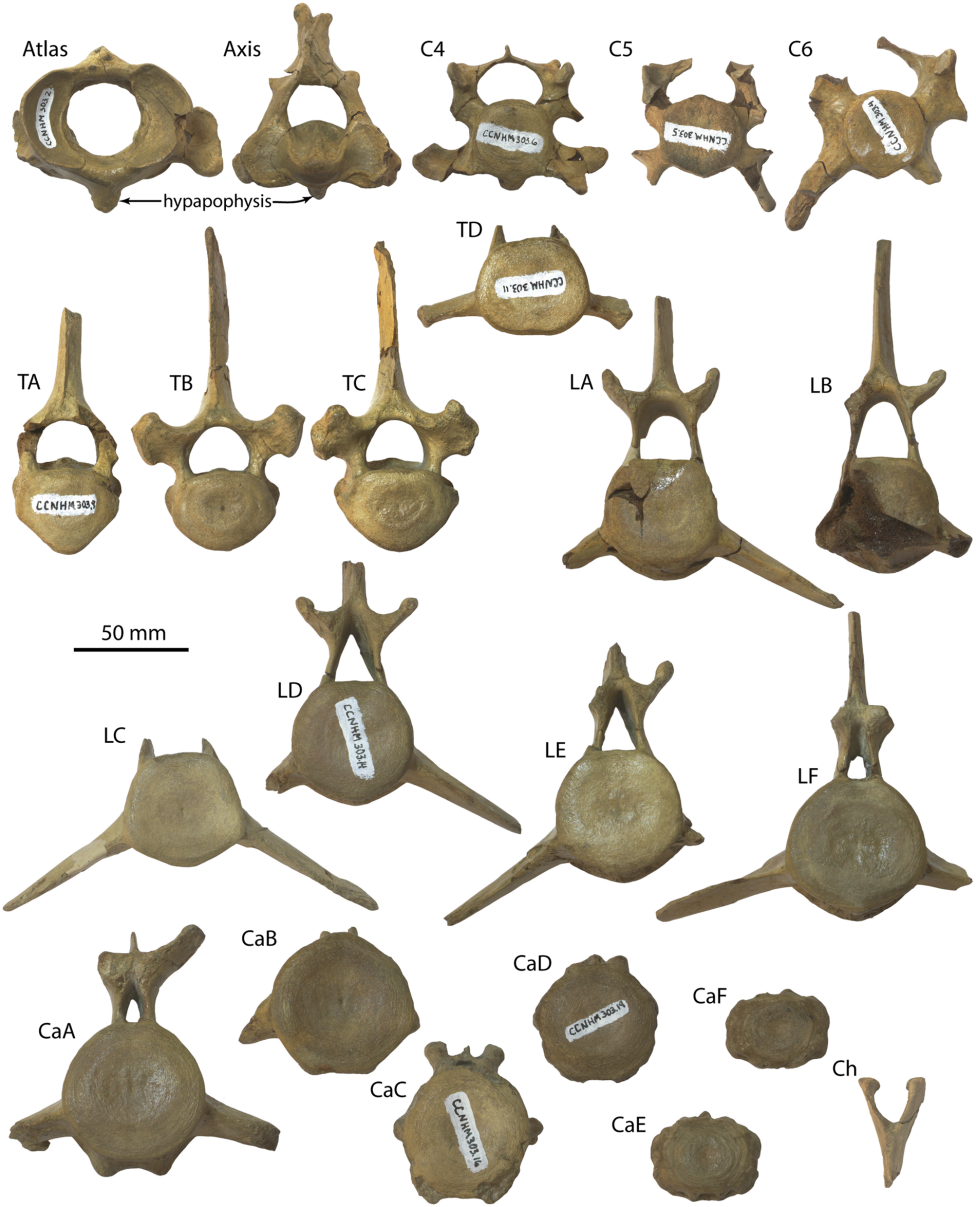
Vertebrae and chevron (Ch) of *Albertocetus meffordorum* (CCNHM 303) in anterior view. Absolute and relative vertebral positions from text.

**Fig 12:**
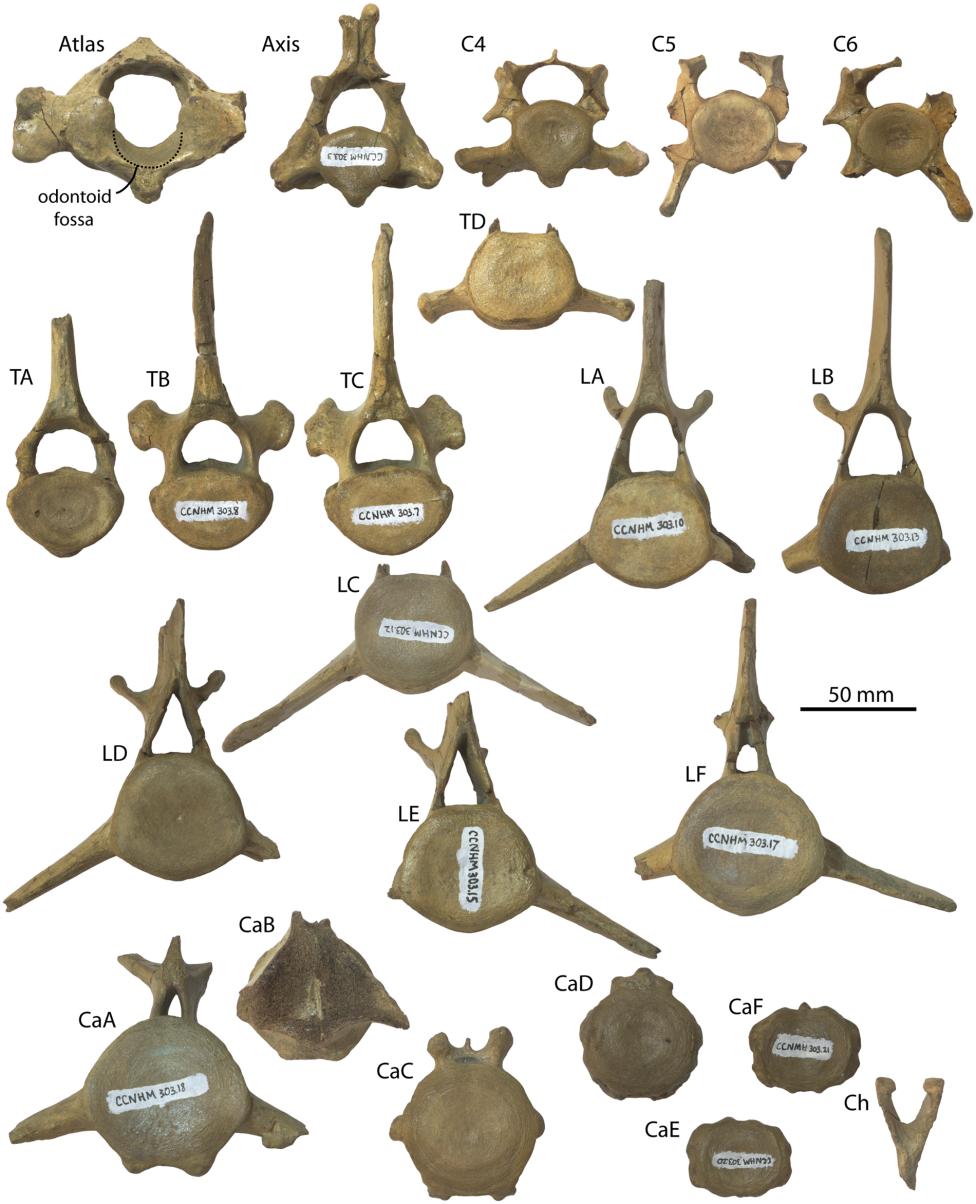
Vertebrae and chevron (Ch) of *Albertocetus meffordorum* (CCNHM 303) in posterior view. Absolute and relative vertebral positions from text.

**Fig 13:**
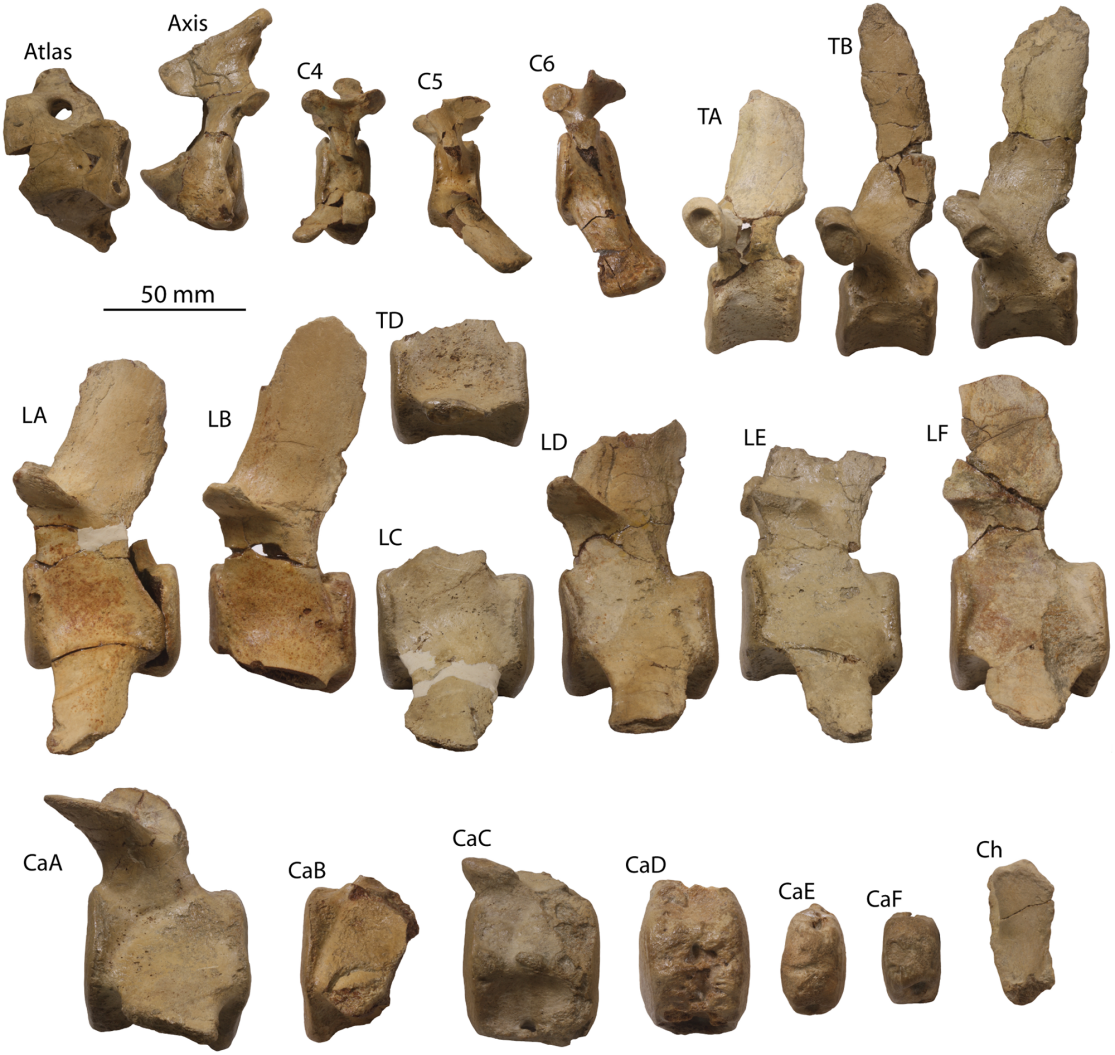
Vertebrae and chevron (Ch) of *Albertocetus meffordorum* (CCNHM 303) in left lateral view. Absolute and relative vertebral positions from text. C6, LE, and LF, are photographed in right lateral view and shown reversed for consistency.

Excluding the transverse process and hypapophysis, the atlas is oval in anterior view and slightly wider than high (Figs 10A–10D and 11–13; Table 4). The vertebral foramen is nearly circular in anterior view, and posteriorly the dorsomedial corners of the axial articular facets intrude into the foramen and divide it into a neural (dorsal) and odontoid (ventral) portion. A large, posterolaterally directed transverse process is present, deepens laterally, and bears dorsal and ventral tubercles. The transverse process is positioned near the posterior margin of the vertebra and bears a shallow fossa on its anterior side that is continuous with a large and circular lateral vertebral foramen. The neural spine is developed as a low tubercle; posteriorly, a flat crescent-shaped facet is developed which seems to receive the articular facet at the anterior apex of the neural spine of the axis (Fig 10B). A deep odontoid fossa is present as is a posteriorly positioned and posteroventrally oriented hypapophysis.

**Table 4:** Atlas and axis vertebra measurements of *Albertocetus meffordorum* (CCNHM 303).

Measurement	Atlas	Axis
Anterior articular facets, transverse width	69.1	63.2
Posterior articular facets, transverse width	66.6	34.7
Anteroposterior length	47	39
Greatest width across transverse processes	106.6	73.7
Neural canal, transverse width	35.9	24.9
Neural canal, dorsoventral depth	33.5	21
Greatest depth	72.7	85.5

#### Axis

The axis is narrower than the atlas and dorsoventrally higher; the neural spine is tall with a bifurcated apex and sagittal groove on its posterior side (Figs 10E–10H, 11–13; Table 4). The spine is triangular in lateral view and continues anteriorly to articulates with the posterior surface of the neural arch of the atlas; a flattened facet suggests the presence of a synovial joint. This facet, in concert with the facet on the atlas, permits approximately 50–55° of longitudinal rotation of the atlanto-axial joint. The postzygapophyses project posterodorsally and are visible jutting outward from the lamina in anterior and posterior views. The vertebral foramen is nearly circular but with a dorsally convex ventral margin. A strong sagittal ridge is present on the dorsal side of the centrum with longitudinal troughs flanking it on either side. The atlantal articular surface is figure-eight shaped with a prominent odontoid process. The hypapophysis is developed as ventrally projecting ridge that deepens posteriorly. The transverse processes are short, posteroventrally oriented, and bear small tubercles at their apices. Fossae are visible in posterior view on the base of the pedicles. The posterior epiphysis is subcircular with a ventral extension onto the hypapophysis.

#### Posterior cervical vertebrae

Three other cervical vertebrae are present, including C4, C5, and C6 (Figs 11–13; Table 5); vertebral positions are identified based upon the well-preserved skeleton of the archaic odontocete *Mirocetus riabinini* (e.g. Riabinin, 1938), as well as the complete cervical series of *Xenorophus* sp. (CCNHM 168). The vertebra considered to be C4 is an anterior cervical vertebra based on the presence of a hypapophysis, but its anterior epiphysis does not match the posterior epiphysis of the axis, meaning it is the C4 not the C3. The vertebrae considered to be C5 lacks a hypapophysis and is thus posterior to C4, but it lacks the ventrolaterally projecting transverse process of the C6. The C6 is identifiable based on the elongate, ventrolaterally projecting transverse process as well as the absence of a hypapophysis. These vertebrae differ most strongly in the structure of the transverse process; but possess centra similar in size and shape. All share neural arches with delicate lamina, dorsoventrally short and narrow neural spines, and bear anteroposteriorly flattened, transversely wide pedicles. The C6 has an anteroposteriorly longer centrum, but in lateral view, the centrum is distinctly wedge-shaped and narrows dorsally, perhaps contributing to a dorsally concave bend in the cervical series. The C4 and C5 had large transverse foramina between the delicate dorsal and larger ventral transverse processes, and appear to have been laterally open given that the ventral transverse processes are complete. In C4 the transverse process is twisted, subtriangular, and oriented anteroventrally to dorsoposteriorly in lateral view. That process widens laterally and exhibits a concave lateral margin in dorsal view with anterior and posterior apices. Several epiphyses C4-C6are fully fused, smooth, and lack a punctate texture indicative of immaturity; in C5, the anterior epiphysis is lost and the posterior epiphysis is partially fused. Both epiphyses in C6 are incompletely fused, with a fine line crease remaining along the margin. Faint annular sulci are present, but notochordal pits are absent.

**Table 5:** Vertebral measurements of *Albertocetus meffordorum* (CCNHM 303).

Measurement	Transverse width, ant. Art. Facet	Height, ant. Art. Facet	Max. transverse width	Centrum length	Max. dorsoventral depth	Neural canal, transverse width	Neural canal, dorsoventral depth
C4	34.5	35.2	85.8	20.1	62.6	27.8	15.5
C5	35.7	34.6	-	17.9	71.7	30.2	-
C6	34.6	36.7	-	24	-	30.1	18.5
TA	42.4	35.5	74.4	37.1	-	24.9	17.8
TB	42.8	33.2	80	38.8	136	25.2	18.9
TC	50.8	34.6	66.5	41.3	135	23.4	18.2
TD	46.6	39.4	90.5	49.9	-	20	-
LA	44	41.7	137.4	55.9	-	21.3	26.8
LB	46.8	44.8	-	53.4	-	23.5	26
LC	46.1	44.3	154	54.8	-	20.6	-
LD	47.1	46	-	55.8	-	17	25.7
LE	47.8	46.4	-	56.9	-	16.1	28
LF	53.9	51.2	-	58.5	-	8.1	25.8
CaA	53.7	51.8	-	56.9	-	7.2	14.3
CaB	56.7	55.7	-	-	-	6.5	-
CaC	55.8	52.8	63.4	50.4	63.8	7.3	2
CaD	46.3	44.3	52	37	54.7	-	-
CaE	35.7	33.1	47.4	20.6	39.1	-	-
CaF	30.7	28.8	44.9	18.6	34.2	-	-

#### Thoracic vertebrae

Four thoracic vertebrae are preserved (Figs 11–13; Table 5), and cannot be readily identified to a particular position; they are identified as TA, TB, TC, and TD and arrayed into an anteroposterior series. In comparison with *Mirocetus riabinini* [46], TA and TB appear to correspond to T2-T5, whereas TC is similar to T6-8. TD is the posteriormost thoracic (T12 in *Mirocetus*; [46]). All share proportionally small but equidimensional centra, and oval-shaped epiphyses; the anterior epiphysis is slightly transversely narrower than the posterior one. TB and TC have well developed flat-shallowly concave facets for the ribs at the dorsolateral edge of the centrum, anteriorly and posteriorly. The vertebral foramen slightly narrows posteriorly in the sequence from TA-TD. The transverse process and pedicle are different in TD and resemble the lumbar vertebrae; TD possesses a short, ventrally positioned, and posterolaterally directed transverse process and a pedicle that is transversely narrower than TA-TC. TD differs from the lumbars in having a flat articular facet for the rib at the lateral terminus of the transverse process. The neural spines of all thoracics are tall and have a concave anterior margin in lateral view. TB and TC possess spines that are anteroposteriorly broader.

#### Lumbar vertebrae

Seven lumbar vertebrae are preserved, and similar to the thoracic vertebrae, cannot be precisely identified to position and instead are identified as LA, LB, LC, LD, LE, LF, and LG (Figs 11–13; Table 5). All lumbars share dorsoventrally flattened, elongate and ventrolaterally projecting transverse processes; the transverse process becomes anteroposteriorly longer further posterior in the series. The vertebral foramen is wide and subrectangular anteriorly, and further posteriorly in the series it becomes narrower and triangular. Within the anterior lumbars (La-Lc) the centrum is absolutely smaller and increases in size further posteriorly; posteriorly the centra become anteroposteriorly longer and transversely wider (e.g. within Ld-Lf). Posteriorly, the neural spines and pedicles become anteroposteriorly broader. The prezygapophyses are large and knob-like anteriorly and become smaller and positioned closer medially further posteriorly within the lumbar series; in LF the prezygapophyses are just small flanges.

#### Caudal vertebrae

Six caudal vertebrae are preserved, identified as CaA-CaF (Figs 11–13; Table 5); they change dramatically from anterior to posterior along the series. CaA is similar to posterior lumbar LF in sharing an elongate centrum with round epiphyses and ventrolaterally projecting transverse processes, but exhibits anterolaterally sloping prezygapophyses and a rhomboidal neural spine that was likely not as tall. The vertebral foramen is smaller than those in the posterior lumbars. CaA is identifiable as an anterior caudal vertebra (perhaps Ca1) by exhibiting paired ventral tubercles for chevrons. CaB and CaC are smaller than CaA; CaB exhibits a more delicate neural arch and a smaller vertebral foramen, and also shares ventrolaterally projecting transverse processes. CaC and CaD are smaller than CaA and CaB, but have minute neural arches, a reduced vertebral foramen, and a small neural spine; the prezygapophyses are developed as small, anteroposteriorly elongate tubercles rising dorsolaterally from the centrum. The transverse processes of CaC and CaD are reduced to small tubercles. CaC and CaD bear chevron articulations formed as elongate paired ventral ridges pierced by canals for the spinal artery; in CaD the canal for the spinal artery (*sensu* [16]) is incised as a vertical channel on the lateral surface of the centrum. By comparison with the extant pygmy sperm whale *Kogia* [47], CaC and CaD corresponds to Ca9-Ca10 and Ca10-12, respectively. CaE and CaF are subrectangular and small with circular to oval articular surfaces; the neural spine and arch are highly reduced. Canals for the spinal arteries pierce the centrum and transverse processes are completely lacking. CaE and CaF correspond to Ca16-19 of *Kogia* [47].

#### Ribs

**Fig 14:**
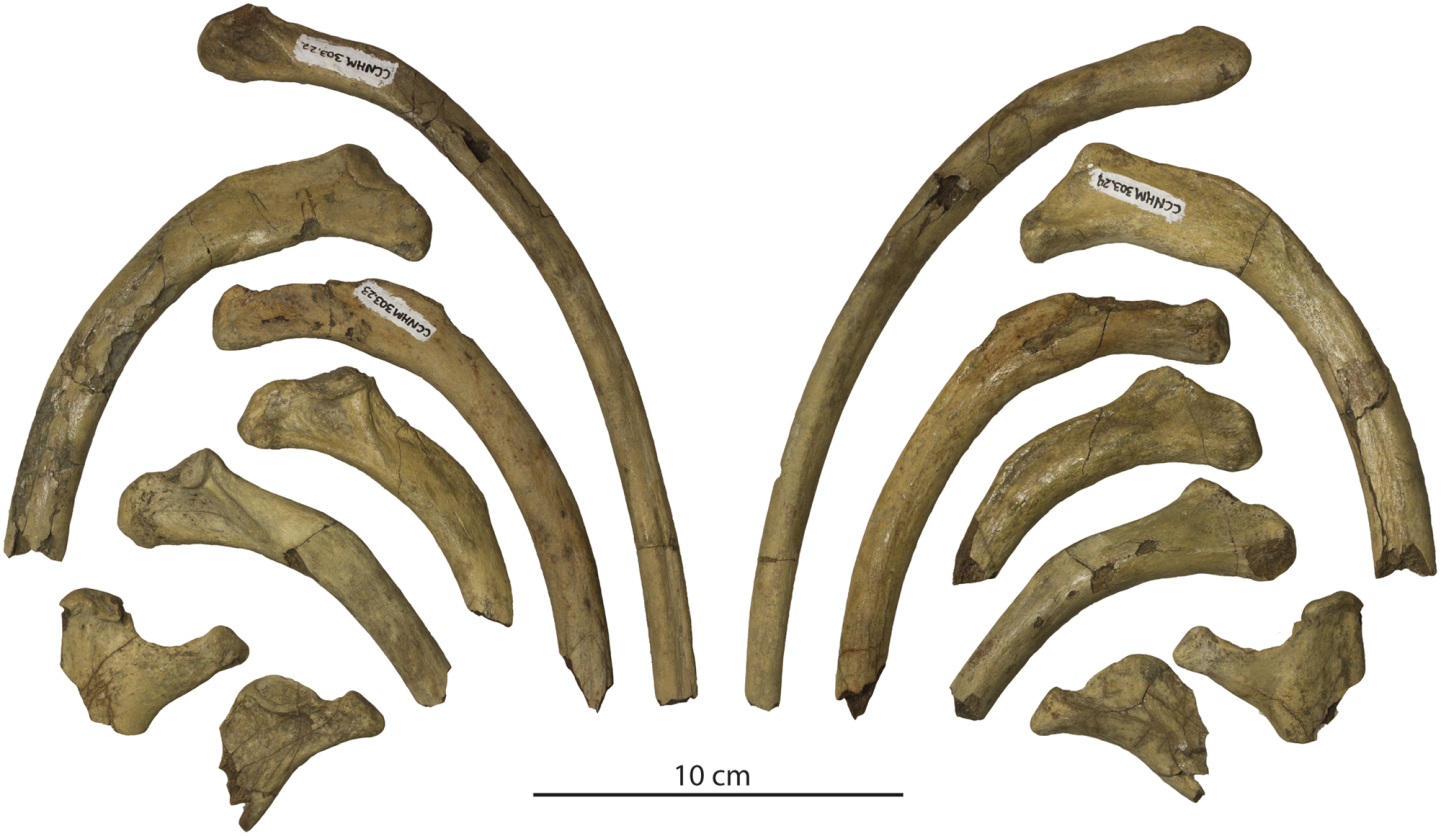
Ribs of *Albertocetus meffordorum* (CCNHM 303) in posterior view (left) and anterior view (right); positions uncertain.

Parts of at least nine ribs are preserved (Fig 14). Judging from narrowly separated capitula and tubercles they are mostly posterior ribs; ribs are gently curving and exhibit circular to oval cross-sections with thick cortices.

#### Chevrons

Parts of two similarly sized chevrons are preserved that include articular tubercles dorsally and a V-shaped body that tapers ventrally (Figs 11–13); they are subrectangular in lateral view. The more complete chevron matches the width of the chevrons on CaA-CaC, suggesting that it is one of the anteriormost chevrons. Relative to the largest and anteriormost caudal vertebra (CaA), the chevron (whilst incomplete) is approximately 3/4 the dorsoventral height of the adjacent caudal centra. By contrast, in the basilosaurids *Dorudon atrox* and *Cynthiacetus peruvianus*, the chevrons are smaller relative to the centrum height, further suggesting an anterior position for these chevrons.

### Taphonomic note

**Fig 15:**
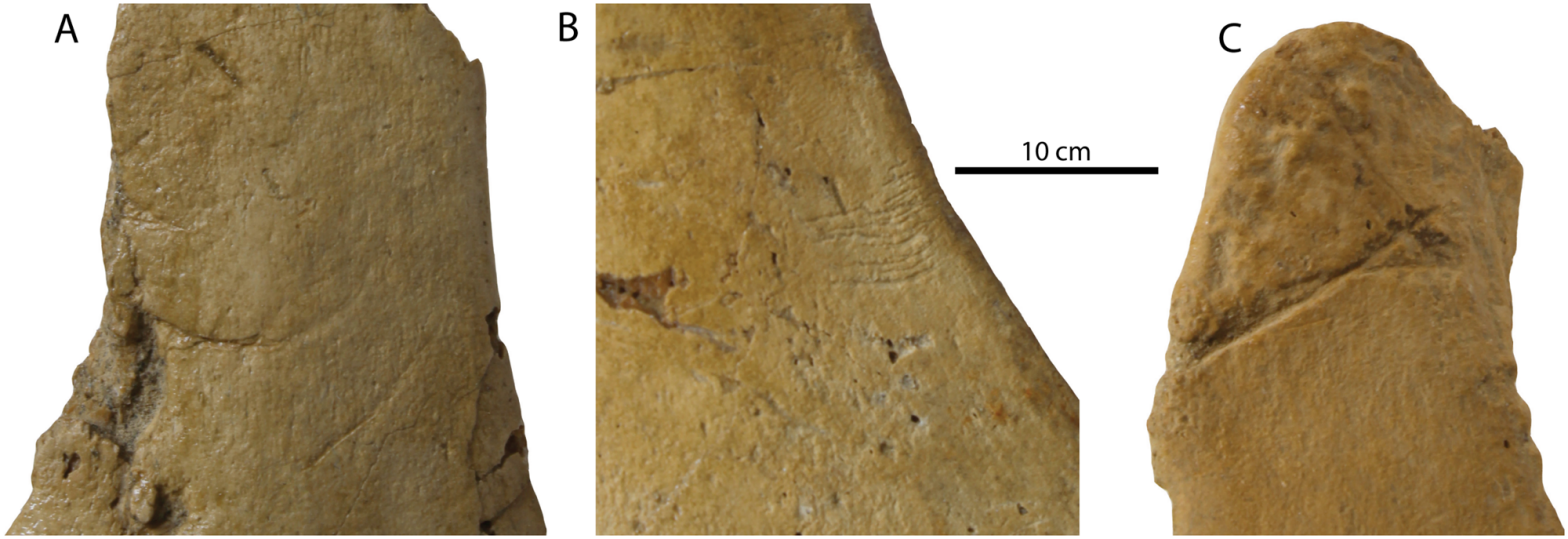
Feeding traces on postcranial elements of *Albertocetus meffordorum* (CCNHM 303). Traces on the transverse process of LE (A), transverse process of LD (B), and chevron (C).

Vertebrae LD, LE, and the more completely preserved chevron preserve several bone modifications (Fig 15). Two types are present: 1) 10–30 mm long linear, arcuate grooves incised into the bone surface (present on the chevron and right transverse process of LE) and 2) a series of short, shallow subparallel scratch marks. These likely represent tooth marks from scavenging sharks (1) and fish, rays, or skates (2). Type 1 correspond most closely to the ichnotaxon *Linichnus* but differ from *Linichnus serratus* in having smooth (rather than serrated) margins [48]; this may prompt the description of a new ichnotaxon for smooth-edged *Linichnus* traces, typically inferred to reflect the tips of shark teeth scraping into bone [49]. Type 2 resemble the short, parallel scratches made by feeding skates (Rajidae) during actualistic feeding experiments [50]; similar traces have been reported on he holotype specimen of the Oligocene baleen whale *Waharoa ruwhenua* [51].

### Encephalization

The finished endocast was shown to have a volume of 478.797cc. If brain density is assumed to be 1.04 g/cc [17], then this would yield a brain mass of 460.4.

**Fig 16:**
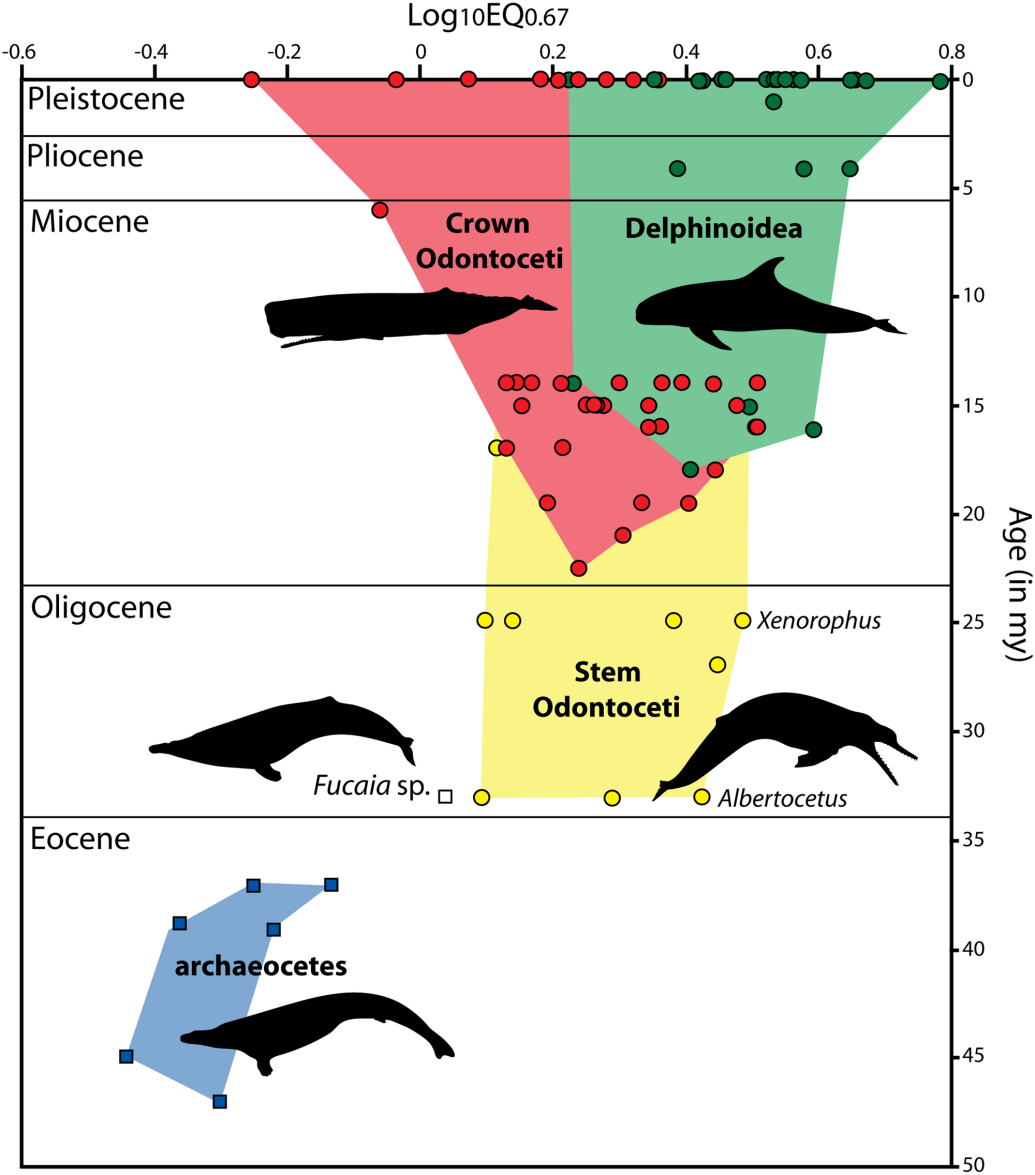
Mean log encephalization quotients (EQ0.67) of cetaceans through time. Archaeocetes are shown as blue squares, stem odontocetes as yellow circles, non-delphinoid crown odontocetes as red circles, and delphinoids as green circles; one mysticete is shown as a white square. Data presented in S1 Table.

Although the digitally rendered endocast closely resembles an isolated brain, endocasts also contain circulatory structures and meninges, even if these structures are not particularly visible. As described in the methods we estimated the volume of adnexia by calculating a linear regression of log(adnexia volume) and log(total endocranial volume) based on data from a recent study [17]. The resulting regression had a r2 of 0.84616 and is described as follows: log (adnexia volume in cc) = 1.7698*log(endocranial volume in cc) - 3.4931. When applied to all fossil odontocetes in the Marino et al dataset [19], it indicates that the estimated brain masses of these extinct taxa should be reduced from 2.1 to 19.6%. For *Albertocetus*, the corrected brain mass would be 443.3 g. The body mass of *Albertocetus*, as inferred by breadth across the occipital condyles [19], is estimated to be 51. 1 kg, which yields an EQ67 of 2.586. The EQ for all other fossil taxa in the Marino et al dataset [19] were recalculated based on these reduced brain masses, but in general this did not affect the overall pattern of change in odontocete encephalization in the fossil record (Fig 16).

We also assessed how alternate estimates for body mass might affect the EQ of Oligocene odontocetes. The width across the bizygomatic processes indicates a total length for *Albertocetus* of 1.82 m and a mass of 72.9 kg, about 71% larger than that estimated from breadth across occipital condyles. Similar discrepancies occur for three other Oligocene odontocetes for which we have bizygomatic width data for, whereas alternative body mass estimates for larger specimens are fairly similar (S1 Table).These higher body masses lead predictably to lower EQ67 values for some Oligocene odontocetes (S1 Table).

## Discussion

### Xenorophids from the Oligocene of North Carolina

The holotype specimen of *Albertocetus meffordorum* (USNM 525001) was collected *ex situ* within a limestone concretion found on Onslow Beach, North Carolina, and presumed to originate from offshore deposits of the upper Oligocene Belgrade Formation [3, 10]. An isolated incomplete, waterworn petrosal (USNM 534010; Fig 8E and 8F) from the same locality was studied by Park et al. [3], who identified the specimen as a xenorophid but were unable to identify any synapomorphies permitting identification as *A*. *meffordorum*. The discovery of a new specimen of *Albertocetus* with a complete petrosal removed from the cranium (CCNHM 303; Fig 7) and additional petrosals (CCNHM 1188, USNM 559392; Fig 8C, 8D, 8G and 8H) from the Belgrade Formation at Belgrade Quarry, North Carolina, invites comparison with the specimen (USNM 534010) reported by Park et al. [3]. Though incomplete, the anterior process of USNM 534010 appears to have been hatchet-shaped with a dorsally concave superior process, like *A*. *meffordorum*, *Echovenator sandersi*, and *Cotylocara macei*. However, USNM 534010 differs from *A*. *meffordorum* and *C*. *macei* in having a broad, shallow suprameatal fossa (Fig 8B, 8D, 8F and 8H) with indistinct margins, and that is anteroposteriorly more elongate than the pars cochlearis, contrasting with the relatively small and deeply excavated pit seen in *Albertocetus meffordorum*, *Cotylocara macei*, and *Xenorophus* sp. A broad suprameatal fossa is a plesiomorphic feature characterizing basilosaurid archaeocetes [45] and some archaic mysticetes including eomysticetids [14] and aetiocetids (*Fucaia buelli*; [44]). Furthermore, the superior process of USNM 534010 is transversely sharp in comparison to the bulbous ridge in *A*. *meffordorum* and other xenorophids. A second petrosal (CCNHM 1188; Fig 8G and 8H) matching the morphology of USNM 534010 (Fig 8E and 8F) and representing the same taxon was recently collected by one of us (RWB) from the Belgrade Formation at the Martin Marietta Aggregates Quarry (colloquially referred to as Belgrade Quarry) in Jones County, North Carolina. These features preclude identification of USNM 534010 and CCNHM 1188 to *A*. *meffordorum*. USNM 534010 and CCNHM 1188 share with *Echovenator* a shallow and anteroposteriorly elongate suprameatal fossa lacking distinct margins, a transversely narrow facial canal, and a posterior bullar facet with an acutely triangular margin (missing in CCNHM 1188). Owing to these features, USNM 534010 and CCNHM 1188 are identifiable as *Echovenator* sp., and in concert with other specimens reported herein, indicate the presence of two xenorophids in the Belgrade Formation of North Carolina. Given that many additional xenorophids have yet to be named from the Ashley and Chandler Bridge formations of South Carolina (CCNHM 171; ChM PV 2758; *Xenorophus* sp.; ChM PV 4746; [1]: Fig 3), it would not be surprising if additional species of xenorophids (and other cetaceans) are discovered from the Belgrade Formation.

### Brain size and EQ in early Odontoceti

An EQ value of 2.586 in *Albertocetus* is within the range of Oligocene odontocetes reported by [19], but a more careful review of their data indicates this is on the high end of EQ expected for a taxon of its age. Marino et al. [19] included seven Paleogene odontocetes in their study, five were assigned to the Chattian (27.0 Ma), one to the Rupelian (33.0 Ma), and one to the late Eocene (38.0 Ma). Based on the USNM collections database, one of the “Chattian” specimens (USNM 256604) and the “late Eocene” specimen (USNM 205491) were collected from the “Toledo Formation”, better known as the Rupelian Alsea Formation [52]; furthermore, one of these specimens, USNM 256604, is actually a small aetiocetid mysticete close in braincase and petrosal morphology to *Fucaia buelli* ([44]; Fig 16). With these more refined ages, *Albertocetus* is the most encephalized of Rupelian odontocetes for which endocast volumes are available (EQ = 1.217–2.586) and near the higher end of values reported for Chattian odontocetes (EQ = 1.234–2.966; Fig 16). Specifically its EQ is exceeded by an unnamed species of *Xenorophus* (ChM PV4266, EQ = 2.966) and another specimen of uncertain affinities (USNM 335502, EQ = 2.718). A similar finding occurs if we compare the EQ values inferred based on model based methods in a phylogenetic context. Montgomery et al. [53] calculated that the node connecting *Xenorophus* to a clade that includes all extant odontocetes had an EQ of 1.6804. Thus on the branch leading to *Xenorophus* there was an inferred change in EQ from 1.6804 to 3.2759. A value of 2.586 for *Albertocetus*, a member of the Xenorophidae, is consistent with this inferred evolutionary change.

One unexpected finding is the difference we found in EQ when using an alternative measures of body mass; specifically bizygomatic width and inferred body length [22, 23]. Among the Oligocene odontocetes for which we have bizygomatic width measured data (either collected by the first author or from a previous study [4]), body size estimated this way are, on average, 136% larger than when estimated using breadth across the occipital condyles. The discrepancy itself seems correlated with size; the bizygomatic widths of the two largest skulls (ChM PV2761, PV2757) actually provide slightly smaller body size estimates (90–91%). Currently we do not have enough measurements to address the significance of these observations, but it is consistent with a previous study that found that occipital condyle breadth typically underestimated body length [22]. If bizygomatic width is a more accurate measure of body mass for Oligocene odontocetes, then the pronounced decreases in body size along the odontocete stem discovered by a previous study [53] could be an artifact. Clearly additional work is needed to improve the accuracy of body size estimate for extinct cetaceans, but that issue is outside of the scope of the current study.

### Ontogeny in Xenorophidae

Ontogenetic studies of extinct cetaceans are rare as they require samples including multiple referred specimens of different ontogenetic stages—whereas many studies emphasizing new taxa and phylogenetic insights have focused on taxa based on single individuals ([12] and references therein). Some studies of extinct mysticetes have examined ontogenetic and intraspecific variation, albeit with small sample sizes [12, 54, 55]. Few comparable studies for extinct Odontoceti exist (but see [56] and [57]). The known sample of *Albertocetus meffordorum* is small (n = 3 specimens with crania), yet informative for the earliest diverging odontocetes.

CCNHM 218 and CCNHM 303 represent different ontogenetic stages owing to a number of differences in cranial suture closure and size of muscle attachment crests. CCNHM 303 differs from 218 in retaining an open parieto-occipital suture and an incompletely closed median frontal suture; these sutures are completely closed in CCNHM 218 (Fig 2). A possible remnant of interparietal in CCNHM 303 (Fig 2A)—absent in 218—further suggests younger ontogenetic status, though partial fusion is suggestive of sexual maturity at minimum [58]. Lastly, CCNHM 303 has cranial crests that are smaller and less prominent than CCNHM 218 (Figs 2–4), including dorsoventrally shallower nuchal crests, anteroposteriorly shorter basioccipital crests, and an anteroposteriorly narrower paroccipital process. Juvenile status of CCNHM 303 is unlikely owing to completely closed intra-occipital sutures [58, 59]. Certain cranial sutures remaining only partially closed in CCNHM 218 (e.g. frontoparietal suture) similarly remain incompletely fused in mature extant odontocetes [58]. Suture closure thus suggests subadult status for CCNHM 303 and adult status for CCNHM 218.

Despite these various cranial features suggesting a younger ontogenetic status for CCNHM 303, nearly all postcranial vertebrae bear completely fused epiphyses (Figs 10–13), with one exception: the anterior epiphysis of C5; similarly, the posterior epiphysis of C5 and both epiphyses of C6 are partially fused. In modern cetaceans, epiphyseal fusion commences anteriorly and posteriorly within the cervical and caudal series and proceeds towards the middle of the column (e.g. thoracics, lumbars), whereas in terrestrial mammals the posterior ossification center is further anterior, within or adjacent to the sacrum [60]. Judging from CCNHM 303, epiphyseal fusion of the posterior cervicals appears to have occurred last in *Albertocetus*. Odontocetes for which published epiphyseal fusion data are available typically lack independent ontogenetic age determinations (but see [61]), but complete fusion in *Balaena mysticetus* and terrestrial mammals does not occur until after sexual maturity [60], perhaps consistent with the skeletally mature vertebral column and skeletally immature cranium of CCNHM 303. Future osteological studies should supplement the work of Moran et al. [60] by recording postcranial epiphyseal fusion as well as scoring cranial suture closure (e.g. [58, 59]). A final point is that these two specimens originate from different members of the Ashley Formation (CCNHM 303, Givhans Ferry Member; CCNHM 218, Runnymede Marl Member), though these two units are of nearly the same age according to ^87^Sr/^86^Sr dates [24].

### Locomotion in early Odontoceti

Studies of locomotor evolution in Cetacea have generally focused on the land-to-sea transition, as superbly documented by archaeocete skeletons from Indo-Pakistan, Egypt, and the southeastern United States [16, 62–67]. Perhaps owing to the relatively modernized postcranial skeletons of most Neogene cetaceans and the dearth of described Oligocene cetaceans with postcrania [12, 14], most studies of locomotor evolution have compared and contrasted various archaeocete grades with modern cetaceans ([16, 68, 69]; but see [70]).

Basilosaurid archaeocetes were the earliest whales restricted to a marine life, though they retained external hindlimbs decoupled from the vertebral column [63]. Posterior thoracic, lumbar, and caudal vertebrae of basilosaurids, all modern and extinct baleen whales, and sperm whales (*Kogia*, *Physeter*) are proportionally similar, permitting dorsoventral undulation along most of the column (= pattern 1 of [70]); these vertebrae are functionally differentiated in all other Odontoceti, with anteroposterior foreshortening of vertebrae stiffening the thoracolumbar series only (= pattern 2 of [70]) or throughout the posterior thoracic, lumbar, and caudal series (= pattern 3 of [70]). Vertebral stiffening serves to limit the amplitude of undulation and enhance swimming speed [70], with extremely foreshortened vertebrae characterizing rapidly swimming delphinids and phocoenids.

**Fig 17:**
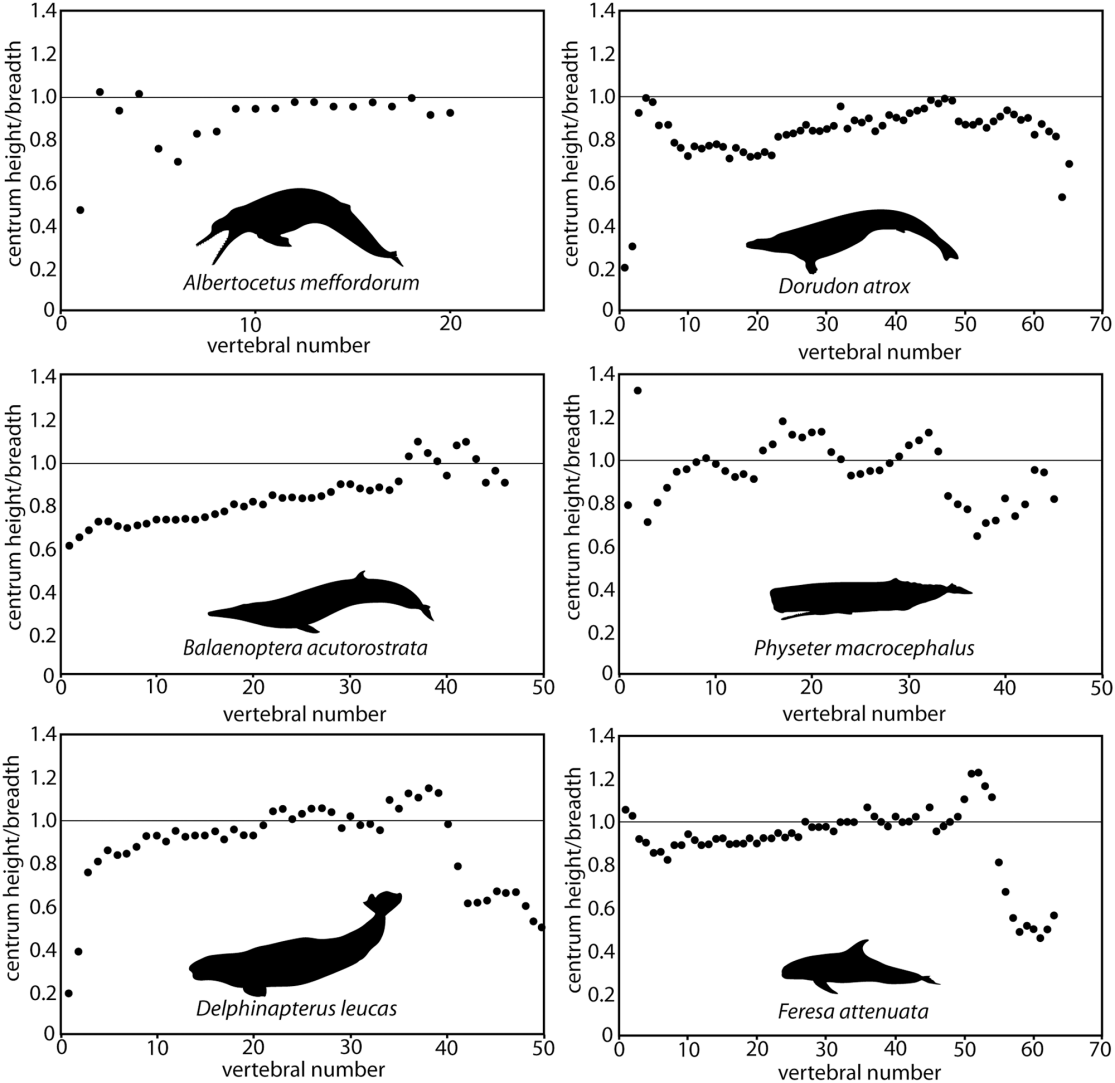
Vertebral proportions (centrum height/centrum breadth) by position of *Albertocetus meffordorum*, *Balaenoptera acutorostrata*, *Delphinapterus leucas*, *Dorudon atrox*, *Feresa attenuata*, and *Physeter macrocephalus*. Data from Omura [72], Omura et al. [73], Yamada [74], Uhen [16], and this study (Table 5).

Rectangular, dorsoventrally shallow terminal caudal vertebrae are osteological correlates of large triangular caudal flukes in modern cetaceans; similarly shaped caudals in basilosaurid whales indicate that flukes are common to all Pelagiceti [16, 70]. In modern cetaceans, the tail immediately anterior to the flukes consists of a streamlined caudal peduncle that is dorsoventrally deep and transversely narrow; extreme narrowness of the body is expressed in anterior and mid-caudal vertebrae that are transversely narrower than they are dorsoventrally deep [16]. Nearly all extant odontocetes (except *Physeter* and *Kogia*) further express anteroposteriorly foreshortened anterior caudal vertebrae, serving to stiffen the peduncle (Patterns 2 and 3 of [70]). Unlike extant cetaceans, the basilosaurid *Dorudon atrox* lacked transversely narrowed anterior caudal vertebrae (Fig 17), suggesting that it lacked a narrowed caudal peduncle [16]. Thus if only extant taxa are considered, it is most parsimonious to infer that a transversely narrow caudal peduncle is a synapomorphy of Neoceti.

The referred skeleton of *Albertocetus meffordorum* (CCNHM 303) possesses the first postcranial skeleton for a xenorophid dolphin (Fig 1C), the earliest diverging clade within Odontoceti [1, 2, 11, 37]. Unsurprisingly, terminal caudal vertebrae (CE and CF) are rectangular and dorsoventrally shallow, indicating the presence of caudal flukes as in all other Pelagiceti (Basilosauridae + Neoceti). Posterior thoracic, lumbar, and anterior caudal vertebrae of CCNHM 303 are all proportionally similar and gradually become longer towards the anterior caudals (Fig 16), paralleling the basilosaurid *Zygorhiza kochii*, the toothed mysticete *Aetiocetus cotylalveus*, extant mysticetes, and sperm whales (Buchholtz [70]). Lack of foreshortening in the anterior caudal vertebrae, and proportional similarity of other 'torso' vertebrae (Fig 17) indicates that *Albertocetus meffordorum* conforms to Pattern 1 of Buchholtz [70]. This is unsurprising, as the early diverging phylogenetic position of *Physeter* and *Kogia* within extant Odontoceti [37] strongly suggests that the Pattern 1 vertebral morphology is the primitive condition amongst Neoceti and stem Odontoceti. Vertebral morphology in *Albertocetus meffordorum* is consistent with mysticete and sperm whale-like locomotion where dorsoventral undulation occurs throughout a flexible vertebral column. This suggests that *Albertocetus* was likely a slower and less efficient swimmer than odontocetes within the clade Synrhina (Delphinoidea + "river dolphins" + Ziphiidae; [37]).

Morphology of the remaining caudal vertebrae is unexpected. Mid-caudal vertebral centra (Ca B, Ca C, Ca D) are nearly equidimensional in shape and lack the transversely narrow profile of modern cetaceans with a caudal peduncle (Figs 11, 12 and 16), and are instead proportionally similar to basilosaurid caudal vertebrae. Although the caudal series of CCNHM 303 is incomplete, this suggests that *Albertocetus meffordorum*—and likely other early diverging odontocetes—primitively lacked a transversely narrow caudal peduncle, similar to the basilosaurid *Dorudon atrox* [16]. The caudal vertebrae of the toothed mysticete *Aetiocetus cotylalveus* are similarly equidimensional ([70]: Fig 7C) possibly indicating the absence of a narrow caudal peduncle, although the distal end of the tail is not represented. Taken together, this data implies that the caudal peduncle evolved independently within Odontoceti and Mysticeti, further highlighting the plasticity of the vertebral skeleton within Cetacea [70]. One caveat to the above suggestion is that only part of the axial skeleton of *Albertocetus meffordorum* is represented, and we can not rule out the presence of a short series of unpreserved vertebrae between Ca D and Ca E that were transversely compressed. Thus additional specimens of archaic Neoceti with even more complete axial skeletons are needed to test our novel hypothesis that a transversely compressed caudal peduncle evolved twice, once in Odontoceti and again in Mysticeti.

## Conclusions

1. New odontocete specimens from the lower Oligocene Ashley Formation of South Carolina include an isolated cranium and a partial skeleton including incomplete cranium with petrotympanics and fragmentary mandible, cervical, thoracic, lumbar, and caudal vertebrae, ribs, and a chevron. These specimens extend the range of *Albertocetus meffordorum* into the early Oligocene.

2. Well-preserved petrosals permit more refined identification of a recently reported petrosal from the upper Oligocene Belgrade Formation of North Carolina as *Echovenator* sp., and permit referral of two additional Belgrade Formation petrosals to *Albertocetus meffordorum* and *Echovenator* sp. Future collecting efforts in North Carolina are expected to yield other cetaceans conspecific with those from the contemporaneous Chandler Bridge Formation of South Carolina.

3. The endocast of *Albertocetus meffordorum* is intermediate in morphology between extant odontocetes and archaeocete whales. Endocast volume indicates that *Albertocetus meffordorum* is the most highly encephalized odontocete from the early Oligocene (EQ = 2.586), well within the range of extant delphinoids, and chronicling a drastic jump in EQ across the Eocene-Oligocene boundary. Further study of appropriate body size estimation is needed to investigate the proposed Eocene-Oligocene explosion in odontocete encephalization.

4. The sample size of *Albertocetus meffordorum* permits the first basic examination of ontogenetic trends in stem Odontoceti. Ontogenetic study of *Albertocetus meffordorum* identifies several sutures of the dorsal braincase and facial region of interest for assessing ontogenetic status in stem Odontoceti (e.g. median parietal suture, frontoparietal suture, frontonasal suture, parieto-occipital suture), to be confirmed with larger samples of undescribed xenorophids (e.g. *Echovenator*, *Xenorophus*). Postcranial epiphyseal fusion is achieved earlier in ontogeny than cranial suture closure in *A*. *meffordorum*.

5. Vertebral proportions indicate that *Albertocetus meffordorum*, like basilosaurids, Mysticeti, and sperm whales, is a "pattern 1" species with no anteroposterior specialization of the vertebral column. This indicates that dorsoventral undulation occurred through the entire flexible lumbocaudal series; this appears to characterize stem odontocetes. Rectangular caudal vertebrae indicate the presence of caudal flukes. Surprisingly, no caudal vertebrae are transversely narrower than tall, suggesting the absence of a transversely narrowed peduncle as in all extant Mysticeti and Odontoceti. Such a feature would imply that the narrow peduncle evolved independently. However, skeletons of stem odontocetes and mysticetes with a more complete caudal series are required to further evaluate this hypothesis.

## Supporting information

S1 Table Endocranial volume and EQ dataset. (XLS)
